# α-Selective Glycosidation of the Rare Sugar d-Tagatofuranose and the Synthesis of α-d-Tagatofuranosylceramide

**DOI:** 10.3390/ijms26178459

**Published:** 2025-08-30

**Authors:** Yui Makura, Akihiro Iyoshi, Makito Horiuchi, Yiming Hu, Masakazu Tanaka, Atsushi Ueda

**Affiliations:** 1 Graduate School of Biomedical Sciences, Nagasaki University, 1-14 Bunkyo-machi, Nagasaki 852-8521, Japanym-hu@nagasaki-u.ac.jp (Y.H.); matanaka@nagasaki-u.ac.jp (M.T.); 2 School of Pharmaceutical Sciences, Nagasaki University, 1-14 Bunkyo-machi, Nagasaki 852-8521, Japan

**Keywords:** d-tagatose, rare sugar, α-selective glycosidation, regioselective protection, furanosyl donor, glycosylceramide, cerebroside

## Abstract

d-Tagatose is a rare sugar that exhibits intriguing biological properties, such as its role as a low-calorie sweetener and its ability to reduce the glycemic response. Consequently, the synthesis of d-tagatose derivatives is a crucial endeavor for the advancement of their functionalities, as well as elucidation of their biological properties. In this study, we present the α-selective glycosidation of a 1,3,4,6-tetra-*O*-benzoylated d-tagatofuranosyl donor with various glycosyl acceptors. In contrast to d-allulose, which is the C3,C4-epimer of d-tagatose and does not exhibit the neighboring group effect, the current d-tagatofuranosyl donor demonstrated significant neighboring group participation, achieving high α-selectivity ratios up to α:β = 99:1. This method was also applicable to the synthesis of α-d-tagatofuranosylceramide, which has potential as a novel functional molecule. Meanwhile, the glycosylation of sterically congested glycosyl acceptors, such as 2-hydroxycumene, resulted in poor α-selectivity, which may be attributed to the interaction with the C1-benzoyloxy group of d-tagatofuranosyl donors in the transition state.

## 1. Introduction

Rare sugars are naturally occurring sugars present in minimal amounts in nature [1]. d-Allulose (d-psicose) and d-tagatose are representatives of rare sugars and have been reported to exhibit significant physiological effects [2,3,4]. For instance, d-allulose has garnered attention because of its biological activities as an antioxidant [5], its role as an inhibitor of *N*-acetylglycosyltransferase [6,7] and α-glucosidase [8,9], its antitumor activity [10], and its GLP-1 releasing activity [11]. d-Tagatose is also an important rare sugar, as it can be used as a low-calorie sweetener and food additive, and has received a Generally Recognized As Safe (GRAS) status certified by the U.S. Food and Drug Administration [12]. In addition, d-Tagatose has remarkable properties [13,14,15], including its ability to significantly lower serum glucose levels following oral glucose intake [16,17,18], and its potential in treating plant diseases [19,20]. Consequently, derivatives of rare sugars are promising compounds for enhancing the functional properties and broadening the diversity of biological properties. On the other hand, glycosylceramides (cerebroside), which are composed of sugar and ceramide moieties, are essential constituents of the human body. Numerous glycosylceramides have been synthesized because of their notable biological activities. For instance, α-galactosylceramide (KRN7000), a ceramide with an α-*O*-glycosidic linkage, is recognized for its ability to activate natural killer T (NKT) cells and stimulate the production of substantial quantities of cytokines [21,22,23]. Recently, several derivatives of KRN7000 have been developed for use as adjuvants in COVID-19 subunit vaccines, immunostimulating agents, and Toll-like receptor 4 (TLR4) agonists [24,25,26]. These examples predominantly involve pyranose as the sugar component, whereas furanosylceramides have rarely been reported. Therefore, d-tagatofuranosylceramides could be promising candidates, although they highlight the necessity for developing stereoselective glycosidation methods. In particular, because d-tagatose is known to exist predominantly in the pyranose form, it is crucial to control these conformers during synthesis [27,28,29].

**Scheme 1:**
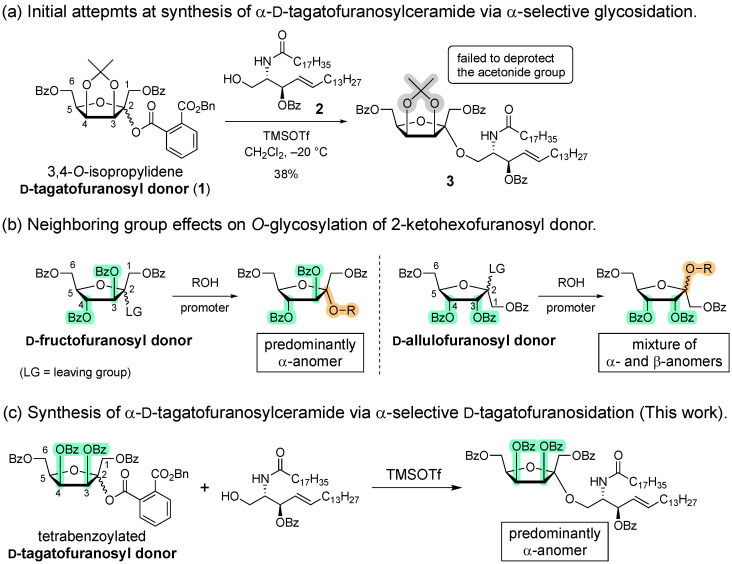
Glycosidation reaction of 2-ketohexofuranoses in previous reports and this work.

We previously reported that the presence of an isopropylidene group at the C3,4-diol position of d-allulofuranosides and d-tagatofuranosides significantly influenced α-selectivity in their glycosidation reactions [30,31,32,33,34,35]. Notably, the *N*- and *S*-glycosidation reactions of a 3,4-*O*-(3-pentylidene)-protected d-allulofuranosyl donor exhibited higher β-selectivities compared to those involving a 3,4-*O*-isopropylidene-protected d-allulofuranosyl donor [36]. Given this context, we initially attempted the glycosidation reaction of the d-tagatofuranosyl donor **1** [34] with ceramide **2** [37], which led to the formation of protected d-tagatofuranosylceramide **3** with a 38% yield and α-selectivity (Scheme 1a). However, attempts to deprotect the acetonide group were unsuccessful due to the concomitant cleavage of the glycosidic bond under acidic conditions. These conditions included the use of aqueous trifluoroacetic acid in CH_2_Cl_2_, *p*-toluenesulfonic acid in MeOH/CH_2_Cl_2_. Consequently, we shifted our focus to a strategy using neighboring acyl group participation, a commonly employed method for stereoselective 1,2-*trans*-glycosylation. Unlike pyranoses, such as d-glucose, this strategy is not consistently effective for 2-ketohexofuranoses. For instance, glycosidation reactions of a d-fructofuranosyl donor with a 3-*O*-benzoyl neighboring group yielded α-glycosides stereoselectively (Scheme 1b) [35,38,39,40]. In contrast, glycosidation of the d-allulofuranosyl donor, the C3-stereoisomer of d-fructofuranose, protected with a 3-*O*-benzoyl group, yielded a mixture of α- and β-glycosides. This outcome is attributed to the C2–C3 eclipsed conformation, which inhibits the participation of the C3-neighboring group effect [39,41]. In this study, we explored the stereoselectivities in the glycosidation of a d-tagatofuranosyl donor, the C4-stereoisomer of d-fructofuranose, protected with 1,3,4,6-tetra-*O*-benzoyl groups, and applied this to the synthesis of α-d-tagatofuranosylceramide using the neighboring group participating strategy (Scheme 1c).

## 2. Results and Discussion

**Scheme 2:**
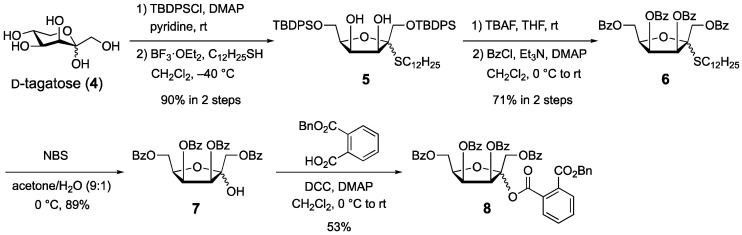
Synthesis of d-tagatofuranosyl donor **8**.

We synthesized d-tagatofuranosyl donor **8** through a six-step process starting from commercially available d-tagatose (**4**; Scheme 2). First, primary alcohols at the C1/C6 positions of d-tagatose were protected using *tert*-butyldiphenylsilyl (TBDPS) groups [42,43,44,45]. Thioglycosidation at the anomeric position was then performed with 1-dodecanethiol and BF_3_·OEt_2_ as the promoter, yielding diol **5** in 90% yield over two steps. Subsequently, the TBDPS groups were removed using tetrabutylammonium fluoride (TBAF), followed by protection with benzoyl chloride in the presence of triethylamine and 4-dimethylaminopyridine (DMAP), resulting in tetrabenzoate **6** in 71% yield over two steps. The thioglycosidic bond in **6** was hydrolyzed by treatment with *N*-bromosuccinimide (NBS) in aqueous acetone, producing hemiacetal **7** in 89% yield. Finally, the anomeric hydroxy group of **7** was esterified with benzyl hydrogen phthalate [46] using *N*,*N′*-dicyclohexylcarbodiimide (DCC) and DMAP, yielding d-tagatofuranosyl donor **8** in 53% yield.

**Scheme 3:**
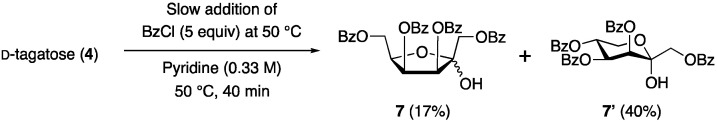
Direct synthesis of tetrabenzoylated d-tagatofuranose **7** along with known pyranose **7′** [47] from d-tagatose.

We also attempted to synthesize glycosyl donor precursor **7** through the direct benzoylation of d-tagatose (Scheme 3). Yamanoi et al. reported that the reaction of d-allulose, the C3, C4-stereoisomer of d-tagatose, with benzoyl chloride preferentially yielded benzoylated furanose in 54% yield along with benzoylated pyranose as a minor byproduct (12%) [39]. However, when applying the same benzoylation conditions as those reported by Yamanoi et al., direct benzoylation of d-tagatose did not result in the formation of furanose **7**. Instead, tetrabenzoylated pyranose **7′** and perbenzoylated products were predominantly formed [47]. Owing to the anomeric effect, d-tagatose as well as its glycoside predominantly adopt the α-d-tagatopyranose form with a ^5^*C*_2_ conformation, as corroborated by the NOESY correlation and X-ray crystallographic analysis [47,48]. When benzoyl chloride was gradually added to the reaction solution at 50 °C, a small amount of tetrabenzoylated d-tagatofuranose **7** (17%, purity: >90%) was isolated after iterative chromatographic separations, along with several byproducts, including pyranose **7′** (40%). Although the process involves many reaction steps, proper protection of d-tagatose followed by thioglycosidation before benzoylation is a necessary step for the synthesis of the benzoylated donor.

Using the prepared d-tagatofuranosyl donor **8**, we investigated its glycosidation reaction with various alcohols. The glycosidation reaction was conducted using 1.5 equivalents of glycosyl acceptors and 1.0 equivalents of trimethylsilyl trifluoromethanesulfonate (TMSOTf) as a promoter in dichloromethane at −20 °C (Table 1). When glycosidation of **8** was performed with primary alcohols such as phenethyl alcohol and 1-dodecanol, the corresponding α-d-tagatofuranosides **9a** and **9b** were stereoselectively obtained in yields of 80% and 72%, respectively (entries 1 and 3). The glycosidation of **8** with phenethyl alcohol at room temperature resulted in a decrease in both the yield and α-selectivity (entry 2). The reaction with 4-nitrobenzyl alcohol yielded **9c** in 81% yield, with an α/β-ratio of 92:8 (entry 4). Glycosidation with secondary alcohols, such as cyclohexanol and isopropanol, resulted in **9d** and **9e** with yields of 78% and 81% and α-selectivities of α/β = 94:6 and 93:7, respectively (entries 5 and 6). However, the use of *p*-methoxyphenol as the glycosyl acceptor led to a lower yield (56% of **9f**) and selectivity (α/β = 89:11, entry 7). The glycosidation reaction with more sterically hindered 2-hydroxycumene further reduced the reaction efficiency, producing only a 15% yield of glycoside **9g** with poor α-selectivity (α/β = 71:29, entry 8). Glycosidation with glycosyl acceptors derived from α-amino acids and sugars were also successful. For instance, the glycosidation of **8** with Fmoc-L-Ser-OMe yielded glycoside **9h** in 76% yield with an α/β ratio of 97:3 (entry 9), and the reactions with methyl 2,3-*O*-isopropylidene-β-d-ribofuranoside and methyl 2,3,4-tri-*O*-benzyl-α-d-glucopyranoside proceeded with high α-selectivity to produce the desired disaccharides **9i** in 82% yield (α/β = 88:12, entry 10) and **9j** in 78% yield (α/β = 96:4, entry 11), respectively. Although the efficiency and α-selectivity of glycosidation depend on the steric hindrance of the acceptor, 3-*O*-benzoylated d-tagatofuranosyl donor **8** proved to be a suitable α-selective glycosyl donor.

**Scheme 4:**
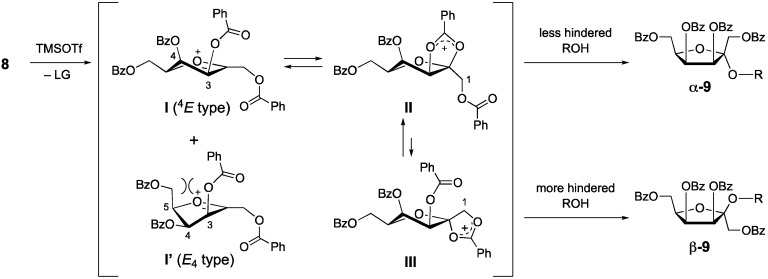
A plausible mechanism for glycosidation of d-tagatofuranosyl donor **8**.

Scheme 4 illustrates a plausible mechanism for the glycosidation reaction of d-tagatofuranosyl donor **8** with alcohols. TMSOTf facilitated the activation of the leaving group (LG = benzyl phthalate) in compound **8**, leading to the formation of oxocarbenium ion intermediates. Owing to the presence of the oxocarbenium ion moiety, these intermediates adopt an envelope-type conformation, either **I** (^4^*E* type) or **I′** (*E*_4_ type) [41,49,50,51]. In the case of conformer **I′**, a 1,3-diaxial interaction occurred between the C3-benzoyloxy group and the C5-benzoyloxymethyl group. Consequently, the conformer **I** is the more favorable intermediate for forming cyclic oxocarbenium intermediate **II**, facilitated by the neighboring group effect of the adjacent C3-benzoyloxy group. As the β-side of the anomeric position in **II** is obstructed by the neighboring group, less sterically hindered alcohols, such as primary alcohols, attack from the α-face of **II**, resulting in the formation of α-anomer **α-9** with high stereoselectivities. Conversely, the approach of more sterically hindered alcohols, such as phenols, may be interrupted by the C1-benzoyloxy group of **II**, which slows down the glycosidation reaction with intermediate **II** and leads to the formation of an alternative cyclic oxocarbenium intermediate **III**. Ultimately, more sterically hindered alcohols partially undergo glycosidation with **III**, yielding undesired **β-9** and resulting in lower α-selectivity in the glycosidation reaction.

**Scheme 5:**
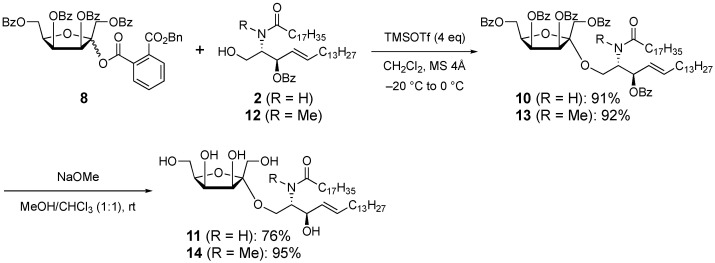
Synthesis of α-d-tagatofuranosylceramide.

As depicted in Scheme 5, we successfully synthesized d-tagatofuranosylceramide **11** using glycosyl donor **8**. The glycosidation reaction between d-tagatofuranosyl donor **8** and ceramide **2** [37] was proceeded α-selectively, yielding the glycoside **10** in 91% yield. In this method, benzoyl protecting groups of **10** were easily removed by a treatment with sodium methoxide in methanol and chloroform and the desired product **11** was obtained in 76% yield. The *N*-methylated d-tagatofuranosylceramide **14** can also be synthesized starting from glycosyl donor **8** and glycosyl acceptor **12**. The glycosidation and saponification of benzoyl groups occurred as similar as **11** to produce *N*-methyl-d-tagatofuranosylceramide **14** in 87% yield in 2 steps.

## 3. Materials and Methods

### 3.1. General Methods

Melting points (Mps) were determined using an AS ONE apparatus ATM-01 and reported without correction (AS ONE Corporation, Osaka, Japan). Optical rotations were measured in CHCl_3_ using a JASCO polarimeter DIP-370 (JASCO Corporation, Tokyo, Japan). IR spectra were measured on an IRAffinity-1 FT-IR spectrophotometer of Shimadzu (Shimadzu Corporation, Kyoto, Japan). ^1^H NMR and ^13^C NMR spectra were measured on Varian NMR System 500PS SN (500 MHz and 126 MHz, Agilent Inc., Santa Clara, CA, USA) or JEOL JNM-ECZ400R (400 MHz and 100 MHz, JEOL Ltd., Tokyo, Japan) spectrometers with chemical shifts (δ) in parts per million (ppm). Tetramethylsilane served as the internal reference (0.00 ppm in CDCl_3_) for the ^1^H NMR spectra, and the central solvent peak was used as the reference (77.0 ppm in CDCl_3_) for the ^13^C NMR spectra. For clarification purposes, the protons of the aldose ring of disaccharides are denoted as 1′, 2′, and so forth. High-resolution mass spectra (HRMS) were collected using a JEOL JMS-T100TD instrument with electrospray ionization (ESI) in time of flight (TOF) mode (JEOL Ltd., Tokyo, Japan). Analytical TLC was conducted using Merck Millipore pre-coated TLC plates, silica gel 60 F_254_ with a layer thickness of 0.25 mm (MilliporeSigma, Burlington, VT, USA). All the moisture sensitive reactions were performed under a nitrogen or an argon atmosphere.

### 3.2. 3-O-Benzoyl-1-O-(1,6-Di-O-benzoyl-3,4-O-isopropylidene-α-d-tagatofuranosyl)-N-stearoyl-d-erythro-sphingosine (***3***)

A mixture of d-tagatofuranosyl donor **1** (72.0 mg, 0.108 mmol) and ceramide **2** (36.1 mg, 0.0539 mmol) was subjected to azeotropic drying through coevaporation with toluene three times, followed by further drying in vacuo. Activated molecular sieves 4 Å (100 mg) and CH_2_Cl_2_ (2 mL) were introduced to this mixture, and the solution was cooled to –20 °C. Subsequently, TMSOTf (9.8 μL, 0.054 mmol) was added to the mixture, and the mixture was stirred at −20 °C for 30 min. After the addition of Et_3_N (0.2 mL), the solvent was removed by evaporation, and the resulting residue was purified by flash column chromatography on silica gel (15% EtOAc in *n*-hexane), yielding product **3** (22.4 mg, 38%) as a white solid. *R*_f_ = 0.69 (40% EtOAc in *n*-hexane). Mp 67–69 °C. [α]^30^_D_: +13.6 (*c* 1.00, CHCl_3_). ^1^H NMR (500 MHz, CDCl_3_) δ: 8.06–7.95 (6H, m, Ar*H*), 7.58–7.47 (3H, m, Ar*H*), 7.47–7.34 (6H, m, Ar*H*), 5.81 (1H, dt, *J*_4′,5′_ = 15.4, *J*_5′,6′_ = 6.8 Hz, H-5′), 5.65 (1H, d, *J*_2′,NH_ = 8.7 Hz, N*H*), 5.53 (1H, dd, *J*_3′,4′_ = 7.2, *J*_2′,3′_ = 5.2 Hz, H-3′), 5.43 (1H, dd, *J*_4′,5′_ = 15.4, *J*_3′,4′_ = 7.2 Hz, H-4′), 4.98 (1H, d, *J*_1a,1b_ = 11.9 Hz, H-1a), 4.90 (1H, dd, *J*_3,4_ = 5.9, *J*_4,5_ = 3.8 Hz, H-4), 4.67 (1H, dd, *J*_6a,6b_ = 11.9, *J*_5,6a_ = 4.2 Hz, H-6a), 4.62 (1H, d, *J*_3,4_ = 5.9 Hz, H-3), 4.48 (1H, dd, *J*_6a,6b_ = 11.9, *J*_5,6b_ = 7.4 Hz, H-6b), 4.43 (1H, dddd, *J*_2′,NH_ = 8.7, *J*_1′a,2′_ = 7.4, *J*_2′,3′_ = 5.2, *J*_1′b,2′_ = 3.8 Hz, H-2′), 4.28 (1H, d, *J*_1a,1b_ = 11.9 Hz, H-1b), 4.27 (1H, ddd, *J*_5,6b_ = 7.4, *J*_5,6a_ = 4.2, *J*_4,5_ = 3.8, H-5), 3.84 (1H, dd, *J*_1′a,1′b_ = 10.0, *J*_1′a,2′_ = 7.4 Hz, H-1′a), 3.75 (1H, dd, *J*_1′a,1′b_ = 10.0, *J*_1′b,2′_ = 3.8 Hz, H-1′b), 1.92 (2H, td, *J*_6′,7′_ = 7.2, *J*_5′,6′_ = 6.8 Hz, H-6′), 1.89–1.78 (2H, m, CH_2_), 1.51 (3H, s, accetonide-CH_3_), 1.47–1.35 (2H, m, CH_2_), 1.34 (3H, s, accetonide-CH_3_), 1.32–1.03 (50H, m, CH_2_), 0.88 (6H, t, *J* = 7.0, CH_3_). ^13^C{^1^H} NMR (126 MHz, CDCl_3_) δ: 173.0, 166.43, 166.39, 165.6, 137.1, 133.4, 133.2, 133.1, 130.2, 129.9 (2C), 129.83 (2C), 129.81, 129.76 (2C), 128.6 (2C), 128.53 (2C), 128.50 (3C), 124.6, 113.5, 107.5, 85.0, 80.1, 78.5, 75.1, 63.0, 59.9, 59.4, 51.5, 36.8, 32.4, 32.1 (2C), 29.9 (5C), 29.83 (5C), 29.81 (2C), 29.76, 29.7, 29.6, 29.52, 29.51 (2C), 29.3 (2C), 29.1, 26.2, 25.8, 24.9, 22.8 (2C), 14.3 (2C). IR (KBr): 2913, 2851, 1721, 1643, 1558 cm^−1^. HRMS (ESI) *m*/*z*: [M + Na]^+^ calcd for C_66_H_97_NO_11_Na, 1102.6959; found, 1102.6961.

### 3.3. (1-Dodecyl) 1,6-Di-O-(tert-butyldiphenylsilyl)-2-thio-d-tagatofuranoside (***5***)

In a solution of d-tagatose (2.00 g, 11.1 mmol) dissolved in pyridine (74 mL), TBDPSCl (8.66 mL, 33.3 mmol) and DMAP (269 mg, 2.22 mmol) were introduced at ambient temperature. The reaction mixture was stirred for three days at the same temperature prior to quenching with 1 M HCl. The aqueous layer was extracted with EtOAc three times, which was washed with sat. NaHCO_3_ aq (twice) and with brine (twice). After drying over anhydrous MgSO_4_ and concentrating under vacuum, the residue was purified using a short pad of silica gel (*n*-hexane, followed by 20% EtOAc in *n*-hexane) to yield crude 1,6-di-*O*-(*tert*-butyldiphenylsilyl)-d-tagatofuranose, which was utilized in the next step without further purification. The above crude product, 1-dodecanethiol (5.32 mL, 22.2 mmol) and activated powdered molecular sieves 4 Å (7.00 g) in CH_2_Cl_2_ (56 mL) were stirred at room temperature for 20 min. Subsequently, BF_3_·OEt_2_ (2.74 mL, 22.2 mmol) was introduced to the mixture at −40 °C, and the mixture was stirred for an additional 15 min at the same temperature. The reaction mixture was quenched by adding Et_3_N (7.0 mL), and the resulting suspension was filtered through a Celite pad. After concentration under vacuum, the residue was purified by flash column chromatography on silica gel (20% EtOAc in *n*-hexane) to afford desired product **5** (8.36 g, 90% in two steps) as colorless oil. *R*_f_ = 0.67 (40% EtOAc in *n*-hexane). ^1^H NMR (400 MHz, CDCl_3_) δ for the major isomer: 7.77–7.65 (8H, m, Ar*H*), 7.49–7.28 (12H, m, Ar*H*), 4.30 (1H, ddd, *J*_4,4-OH_ = 10.4, *J*_3,4_ = 4.5, *J*_4,5_ = 3.6 Hz, H-4), 4.23 (1H, dd, *J*_3,3-OH_ = 9.7, *J*_3,4_ = 4.5 Hz, H-3), 4.22 (1H, ddd, *J*_5,6b_ = 5.8, *J*_5,6a_ = 5.4, *J*_4,5_ = 3.6 Hz, H-5), 4.03 (1H, dd, *J*_6a,6b_ = 10.7, *J*_5,6a_ = 5.4 Hz, H-6a), 3.93 (1H, dd, *J*_6a,6b_ = 10.7, *J*_5,6b_ = 5.8 Hz, H-6b), 3.89 (1H, d, *J*_1a,1b_ = 10.8 Hz, H-1a), 3.84 (1H, d, *J*_1a,1b_ = 10.8 Hz, H-1b), 3.46 (1H, d, *J*_4,4-OH_ = 10.4 Hz, 4-O*H*), 3.46 (1H, d, *J*_3,3-OH_ = 9.7 Hz, 3-O*H*), 1.44–1.13 (23H, m), 1.11–0.97 (17H, m), 0.92–0.83 (3H, m). ^13^C{^1^H} NMR (100 MHz, CDCl_3_) δ for the major isomer: 135.7 (2C), 135.6 (2C), 135.5 (2C), 133.2, 133.0, 132.1, 131.8, 130.1, 129.9, 129.7 (2C), 127.9 (2C), 127.8 (2C), 127.73 (3C), 127.71 (3C), 93.2, 80.6, 79.2, 72.2, 65.1, 61.8, 31.9, 30.3, 29.62 (2C), 29.56, 29.50, 29.3, 29.1, 29.0, 28.9, 26.7 (3C), 26.6 (3C), 22.7, 19.2, 19.0, 14.1. IR (KBr): 3393, 3073, 2957, 2928, 2857 cm^−1^. HRMS (ESI) *m*/*z*: [M + Na]^+^ calcd for C_50_H_72_O_5_SSi_2_Na, 863.4537; found, 863.4544.

### 3.4. (1-Dodecyl) 1,3,4,6-Tetra-O-benzoyl-2-thio-d-tagatofuranoside (***6***)

To a solution of **5** (7.31 g, 8.96 mmol) in THF (87 mL) was added tetra-*n*-butylammonium fluoride (TBAF; 1 M in THF, 34.8 mL, 34.8 mmol) solution at room temperature. After stirring at room temperature for 1 d, the ice-cold reaction mixture was quenched by adding Dowex 50W × 8 (21.4 g), CaCO_3_ (7.14 g), and MeOH (51 mL) [52], which was further stirred for 10 min at 0 °C and for 30 min at room temperature. The reaction mixture was filtered through a Celite pad and the filter cake was washed with MeOH. After concentration in vacuo, the residue was passed through a short plug of silica gel (2 cm, CHCl_3_, then 10% MeOH in *n*-hexane) to give crude (1-dodecyl) 2-thio-d-tagatofuranoside (**S2**) as a colorless oil [*R*_f_ = 0.37 (EtOAc)]. To a solution of the above tetraol **S2**, Et_3_N (24.3 mL, 174 mmol), and DMAP (531 mg, 4.35 mmol) in CH_2_Cl_2_ (87 mL) was added benzoyl chloride (BzCl; 10.1 mL, 86.9 mmol) dropwise at 0 °C and then the mixture was stirred at room temperature for 1 d. The reaction mixture was quenched by adding MeOH (10 mL) at 0 °C and stirred for a further 40 min at room temperature. After removal of MeOH by evaporation, the residue was partitioned into 1 M HCl aq (30 mL) and EtOAc (30 mL) layers. The aqueous residue extracted with EtOAc (40 mL) three times, and the combined organic layers were washed with water, sat. NaHCO_3_ aq, and brine (20 mL each), dried over anhydrous MgSO_4_ and concentrated under vacuum. The crude material was purified by flash column chromatography on silica gel (5% then 20% EtOAc in *n*-hexane) to afford tetrabenzoate **6** (4.84 g, 71% in two steps, α:β = 17:1) as a pale-yellow oil. *R*_f_ = 0.54 (30% EtOAc in *n*-hexane). *R*_f_ = 0.51 (30% EtOAc in *n*-hexane). ^1^H NMR (400 MHz, CDCl_3_) δ for the major isomer: 7.99–7.94 (2H, m, Ar*H*), 7.94–7.89 (2H, m, Ar*H*), 7.87–7.81 (4H, m, Ar*H*), 7.56–7.42 (4H, m, Ar*H*), 7.41–7.35 (2H, m, Ar*H*), 7.34–7.28 (2H, m, Ar*H*), 7.28–7.20 (4H, m, Ar*H*), 6.24 (1H, dd, *J*_4,5_ = 6.0, *J*_3,4_ = 5.5 Hz, H-4), 5.89 (1H, d, *J*_3,4_ = 5.5 Hz, H-3), 4.96 (1H, ddd, *J*_5,6a_ = 6.6, *J*_4,5_ = 6.0, *J*_5,6b_ = 5.6 Hz, H-5), 4.85 (1H, d, *J*_1a,1b_ = 11.9 Hz, H-1a), 4.73 (1H, dd, *J*_6a,6b_ = 11.6, *J*_5,6a_ = 6.6 Hz, H-6a), 4.68 (1H, d, *J*_1a,1b_ = 11.9 Hz, H-1b), 4.68 (1H, dd, *J*_6a,6b_ = 11.6, *J*_5,6b_ = 5.6 Hz, H-6b), 2.75 (2H, t, *J* = 7.3 Hz, SC*H*_2_), 1.70–1.56 (2H, m, SCH_2_C*H*_2_), 1.43–1.32 (2H, m, SCH_2_CH_2_C*H*_2_), 1.32–1.19 (16H, m), 0.88 (3H, t, *J* = 7.0 Hz, CH_3_). ^13^C{^1^H} NMR (100 MHz, CDCl_3_) δ for the major isomer: 166.1, 165.8, 165.1, 164.7, 133.4 (2C), 133.1, 133.0, 129.70 (3C), 129.67 (2C), 129.65 (3C), 129.5 (2C), 128.7, 128.6, 128.4 (4C), 128.3 (2C), 128.2 (2C), 92.5, 77.0, 76.0, 72.6, 62.9, 62.5, 31.9, 29.7, 29.62, 29.59, 29.54, 29.49, 29.3, 29.1, 29.0, 28.6, 22.7, 14.1. IR (KBr): 3065, 2955, 2924, 2853, 1726, 1603 cm^−1^. HRMS (ESI) *m*/*z*: [M + Na]^+^ calcd for C_46_H_52_O_9_SNa, 803.3230; found, 803.3233.

### 3.5. 1,3,4,6-Tetra-O-benzoyl-d-tagatofuranose (***7***)

Synthesis from **6**: To a solution of **6** (4.78 g, 6.13 mmol) in a mixture of acetone (55 mL) and H_2_O (6 mL) was added *N*-bromosuccinimide (2.18 g, 12.2 mmol) at 0 °C and the reaction was stirred at 0 °C for 15 min. An additional *N*-bromosuccinimide (1.09 g, 6.13 mmol) was added to the reaction, which was further stirred at 0 °C for 15 min. After quenching the reaction by adding sat. NaHCO_3_ aq, the solvent was removed under reduced pressure. The reaction mixture was extracted with EtOAc three times and dried over anhydrous MgSO_4_. The crude material obtained after evaporation was purified by flash column chromatography on silica gel (10% then 30% EtOAc in *n*-hexane) to afford the desired product **7** (3.23 g, 89%, α:β = 1.1:1). Synthesis from **4**: To a solution of d-tagatose (901 mg, 5.00 mmol) in pyridine (15 mL), benzoyl chloride (2.90 mL, 25.0 mmol) was added at 50 °C over 15 min using a syringe pump, and the mixture was stirred for an additional 25 min. The reaction was quenched by the addition of MeOH (3 mL) and stirring at room temperature for 10 min. After evaporating pyridine, the residue was dissolved in 50% EtOAc in *n*-hexane and washed with 1 M HCl aq and water. The organic phase was dried over anhydrous MgSO_4_ and concentrated under vacuum. The residue was purified by flash column chromatography on silica gel (30% EtOAc in *n*-hexane, iterative), yielding compound **7** (495 mg, 17%) along with **7′** [28] (1.18 g, 40%). Colorless oil. *R*_f_ = 0.23 (30% EtOAc in *n*-hexane). ^1^H NMR (400 MHz, CDCl_3_) δ: 8.08–7.82 (8H, m, Ar*H*), 7.62–7.45 (4H, m, Ar*H*), 7.45–7.26 (8H, m, Ar*H*), 6.21 (0.5H, dd, *J*_4,5_ = 5.9, *J*_3,4_ = 5.1 Hz, α-H-4), 6.17 (0.5H, dd, *J*_3,4_ = 5.3, *J*_4,5_ = 5.0 Hz, β-H-4), 5.93 (0.5H, d, *J*_3,4_ = 5.1 Hz, α-H-3), 5.85 (0.5H, d, *J*_3,4_ = 5.3 Hz, β-H-3), 5.01 (0.5H, ddd, *J*_5,6a_ = 6.9, *J*_4,5_ = 5.9, *J*_5,6b_ = 5.6 Hz, α-H-5), 4.89 (0.5H, dd, *J*_6a,6b_ = 11.3, *J*_5,6a_ = 7.1 Hz, β-H-6a), 4.86 (0.5H, d, *J*_1a,1b_ = 11.8 Hz, H-1), 4.81 (0.5H, dt, *J*_5,6a_ = 7.1, *J*_4,5_ = *J*_5,6b_ = 5.0 Hz, β-H-5), 4.70 (0.5H, dd, *J*_6a,6b_ = 11.6, *J*_5,6a_ = 6.9 Hz, α-H-6a), 4.65–4.56 (2H, m, H-1a, 1b, α-H-6b, β-H-6b), 4.54 (0.5H, d, *J*_1a,1b_ = 11.7 Hz, H-1), 4.00 (1H, br s, O*H*). ^13^C{^1^H} NMR (100 MHz, CDCl_3_) δ: 166.8 (0.5C), 166.4 (0.5C), 166.1 (0.5C), 165.9 (0.5C), 165.1 (0.5C), 165.0 (0.5C), 164.9 (0.5C), 164.8 (0.5C), 133.7 (0.5C), 133.6 (0.5C), 133.57 (0.5C), 133.55 (0.5C), 133.3 (0.5C), 133.2, 133.1 (0.5C), 129.8–129.7 (8C), 129.5 (0.5C), 129.4 (0.5C), 129.3 (0.5C), 129.1 (0.5C), 128.7–128.2 (10C), 103.7 (0.5C), 101.7 (0.5C), 76.5 (0.5C), 76.2 (0.5C), 75.9 (0.5C), 72.3 (0.5C), 72.1 (0.5C), 71.5 (0.5C), 66.1 (0.5C), 65.2 (0.5C), 63.2 (0.5C), 63.1 (0.5C). IR (KBr): 3437, 3065, 3032, 2965, 1732 cm^−1^. HRMS (ESI) *m*/*z*: [M + Na]^+^ calcd for C_34_H_28_O_10_Na, 619.1580; found, 619.1579.

### 3.6. 1,3,4,6-Tetra-O-benzoy-d-tagatofuranosyl phthalate (***8***)

DCC (158 mg, 0.758 mmol) and DMAP (31.3 mg, 0.256 mmol) were added to a mixed solution of **7** (153 mg, 0.256 mmol) and monobenzyl phthalate (230 mg, 0.898 mmol) in CH_2_Cl_2_ (3 mL) at 0 °C. The reaction mixture was subsequently allowed to gradually reach room temperature over 3 h. The reaction mixture was filtered through a pad of Celite and the filter cake was rinsed by CH_2_Cl_2_. The filtrate was washed sequentially with 5% Na_2_CO_3_ aqueous solution, water, and brine. The organic phase was dried over MgSO_4_ and concentrated under vacuum to give a residue, which was purified by flash column chromatography on silica gel eluted with 5% EtOAc in *n*-hexane to yield compound **8** (114 mg, 53%). Colorless oil. *R*_f_ = 0.57 (5% Et_2_O in CHCl_3_). [α]^23^_D_: +39.8 (*c* 1.00, CHCl_3_). ^1^H NMR (400 MHz, CDCl_3_) δ for the major isomer: 7.98–7.84 (8H, m, Ar*H*), 7.84–7.80 (1H, m, Ar*H*), 7.73–7.68 (1H, m, Ar*H*), 7.56–7.45 (6H, m, Ar*H*), 7.43–7.39 (2H, m, Ar*H*), 7.39–7.26 (11H, m, Ar*H*), 6.53 (1H, d, *J* = 5.3 Hz, H-3), 6.20 (1H, dd, *J*_3,4_ = 5.3, *J*_4,5_ = 4.7 Hz, H-4), 5.37 (1H, d, *J* = 12.3 Hz, -C*H*_2_Ph), 5.32 (1H, d, *J* = 12.3 Hz, -C*H*_2_Ph), 5.25 (1H, d, *J*_1a,1b_ = 12.0 Hz, H-1a), 5.20 (1H, ddd, *J*_5,6a_ = 6.4, *J*_5,6b_ = 5.7, *J*_4,5_ = 4.7 Hz, H-5), 4.94 (1H, d, *J*_1a,1b_ = 12.0 Hz, H-1b), 4.70 (1H, dd, *J*_6a,6b_ = 11.7, *J*_5,6a_ = 6.4 Hz, H-6a), 4.64 (1H, dd, *J*_6a,6b_ = 11.7, *J*_5,6b_ = 5.7 Hz, H-6b). ^13^C{^1^H} NMR (100 MHz, CDCl_3_) δ for the major isomer: 166.7, 166.1, 166.0, 165.7, 165.1, 164.4, 135.5, 133.6, 133.5, 133.1, 133.1, 132.5, 131.6, 131.2, 131.0, 129.7 (9C), 129.41, 129.38, 129.3, 129.0, 128.6, 128.5 (4C), 128.4 (4C), 128.29 (2C), 128.3 (2C), 109.3, 78.5, 75.5, 72.3, 67.5, 63.2, 62.4. IR (KBr): 3065, 3032, 2961, 1724, 1601 cm^−1^. HRMS (ESI) *m*/*z*: [M + Na]^+^ calcd for C_49_H_38_O_13_Na, 857.2210; found, 857.2219.

### 3.7. General Procedure for the Glycosidation Described in Table 1

Azeotropic drying with toluene in vacuo was performed on the glycosyl acceptor (0.150 mmol) and glycosyl donor **8** (83.5 mg, 0.100 mmol), resulting in a mixture, which was subsequently dissolved in CH_2_Cl_2_ (2.0 mL) and TMSOTf (18.1 μL, 0.100 mmol) was added at −20 °C (at 0 °C for entry 3). Upon completion of the reaction under the conditions specified in Table 1, Et_3_N (0.1 mL) was added to the reaction mixture, which was stirred at ambient temperature for an additional 5 min. After removing the solvent in vacuo, the residue was purified by flash column chromatography on silica gel (EtOAc/*n*-hexane, and CHCl_3_ if necessary) to yield the corresponding product.

#### 3.7.1. (2-Phenethyl) 1,3,4,6-Tetra-*O*-benzoyl-α-d-tagatofuranoside (**9a**)

According to the general procedure for the glycosidation reaction, compound **9a** (56.3 mg) was obtained from the glycosyl donor **8** (83.5 mg, 0.100 mmol) and phenethyl alcohol (0.0180 mL, 0.150 mmol) in 80% yield as a 99:1 mixture of α- and β-anomers. Colorless oil. Eluent for column: 10% EtOAc in *n*-hexane for the first column and CHCl_3_ for second column. [α]^23^_D_: +92.7 (*c* 1.00, CHCl_3_). ^1^H NMR (500 MHz, CDCl_3_) δ: 7.96–7.87 (6H, m, Ar*H*), 7.81–7.73 (2H, m, Ar*H*), 7.57–7.47 (3H, m, Ar*H*), 7.47–7.42 (1H, m, Ar*H*), 7.40–7.30 (6H, m, Ar*H*), 7.28–7.26 (3H, m, Ar*H*), 7.26–7.19 (3H, m, Ar*H*), 7.19–7.13 (1H, m, Ar*H*), 5.97 (1H, dd, *J*_4,5_ = 6.6, *J*_3,4_ = 5.2 Hz, H-4), 5.89 (1H, d, *J*_3,4_ = 5.2 Hz, H-3), 4.89 (1H, d, *J*_1a,1b_ = 12.2 Hz, H-1a), 4.57 (1H, dd, *J*_6a,6b_ = 11.5, *J*_5,6a_ = 6.8 Hz, H-6a), 4.44 (1H, dd, *J*_6a,6b_ = 11.5, *J*_5,6b_ = 5.8 Hz, H-6b), 4.44 (1H, d, *J*_1a,1b_ = 12.2 Hz, H-1b), 4.13 (1H, ddd, *J*_5,6a_ = 6.8, *J*_4,5_ = 6.6, *J*_5,6b_ = 5.8 Hz, -5H), 3.99–3.83 (2H, m, OC*H*_2_CH_2_Ph), 2.94–2.83 (2H, m, C*H*_2_Ph). ^13^C{^1^H} NMR (126 MHz, CDCl_3_) δ: 166.0, 165.7, 165.0, 164.7, 139.0, 133.4, 133.3, 133.1, 133.0, 129.7 (2C), 129.62 (6C), 129.57, 129.3, 129.0 (2C), 128.9, 128.7, 128.5 (2C), 128.4 (2C), 128.3 (4C), 128.2 (2C), 126.3, 106.0, 75.7, 75.4, 72.0, 63.0, 62.9, 59.7, 36.2. IR (KBr): 3063, 2957, 1724 cm^−1^. HRMS (ESI) *m*/*z*: [M + Na]^+^ calcd for C_42_H_36_O_10_Na, 723.2206; found, 723.2209.

#### 3.7.2. (1-Dodecyl) 1,3,4,6-Tetra-*O*-benzoyl-α-d-tagatofuranoside (**9b**)

According to the general procedure for the glycosidation reaction, compound **9b** (55.1 mg) was obtained from the glycosyl donor **8** (83.5 mg, 0.100 mmol) and 1-dodecanol (27.9 mg, 0.150 mmol) in 72% yield as a 99:1 mixture of α- and β-anomers. Colorless oil. Eluent for column: 5% EtOAc in *n*-hexane for the first column and CHCl_3_ for second column. [α]^23^_D_: +79.5 (*c* 1.00, CHCl_3_). ^1^H NMR (500 MHz, CDCl_3_) δ: 7.99–7.90 (6H, m, Ar*H*), 7.83–7.77 (2H, m, Ar*H*), 7.55–7.48 (3H, m, Ar*H*), 7.48–7.42 (1H, m, Ar*H*), 7.40–7.31 (6H, m, Ar*H*), 7.28–7.21 (2H, m, Ar*H*), 6.18 (1H, dd, *J*_4,5_ = 6.6, *J*_3,4_ = 5.2 Hz, H-4), 5.94 (1H, d, *J*_3,4_ = 5.2 Hz, H-3), 4.93 (1H, d, *J*_1a,1b_ = 12.3 Hz, H-1a), 4.84 (1H, ddd, *J*_5,6a_ = 6.7, *J*_4,5_ = 6.6, *J*_5,6b_ = 5.6 Hz, H-5), 4.71 (1H, dd, *J*_6a,6b_ = 11.5, *J*_5,6a_ = 6.7 Hz, H-6a), 4.58 (1H, dd, *J*_6a,6b_ = 11.5, *J*_5,6b_ = 5.6 Hz, H-6b), 4.48 (1H, d, *J*_1a,1b_ = 12.3 Hz, H-1b), 3.71 (1H, dt, *J* = 9.0, 6.5 Hz, OC*H*HCH_2_-), 3.61 (1H, dt, *J* = 9.0, 6.4 Hz, OCH*H*CH_2_-), 1.62–1.54 (2H, m, OCH_2_C*H*_2_-), 1.39–1.31 (2H, m, OCH_2_CH_2_C*H*_2_-), 1.30–1.19 (16H, m, C*H*_2_), 0.88 (3H, t, *J* = 6.9 Hz, C*H*_3_). ^13^C{^1^H} NMR (126 MHz, CDCl_3_) δ: 166.1, 165.7, 165.2, 164.7, 133.40, 133.36, 133.1, 133.0, 129.71 (2C), 129.67 (2C), 129.6 (4C), 129.5, 129.4, 128.9, 128.7, 128.5 (2C), 128.31 (2C), 128.29 (2C), 128.25 (2C), 106.2, 75.9, 75.5, 72.2, 63.3, 62.0, 59.6, 31.9, 29.7, 29.6, 29.6, 29.6, 29.6, 29.4, 29.3, 26.2, 22.7, 14.1. IR (KBr): 3063, 2926 1728 cm^−1^. HRMS (ESI) *m*/*z*: [M + Na]^+^ calcd for C_46_H_52_O_10_Na, 787.3458; found, 787.3454.

#### 3.7.3. (4-Nitrobenzyl) 1,3,4,6-Tetra-*O*-benzoyl-α-d-tagatofuranoside (**9c**)

According to the general procedure for the glycosidation reaction, compound **9c** (59.4 mg) was obtained from the glycosyl donor **8** (83.5 mg, 0.100 mmol) and 4-nitrobenzyl alcohol (23.0 mg, 0.150 mmol) in 81% yield as a 92:8 mixture of α- and β-anomers. Colorless oil. Eluent for column: 10% EtOAc in *n*-hexane for the first column and CHCl_3_ for second column. [α]^23^_D_: +71.7 (*c* 1.00, CHCl_3_). ^1^H NMR (500 MHz, CDCl_3_) δ: 8.16–8.09 (2H, m, Ar*H*), 7.99–7.92 (4H, m, Ar*H*), 7.89–7.80 (4H, m, Ar*H*), 7.56–7.47 (6H, m, Ar*H*), 7.41–7.32 (6H, m, Ar*H*), 7.30–7.26 (2H, m, Ar*H*), 6.18 (1H, dd, *J*_4,5_ = 6.3, *J*_3,4_ =5.3 Hz, H-4), 6.08 (1H, d, *J*_3,4_ = 5.3 Hz, H-3), 5.07 (1H, d, *J*_1a,1b_ = 12.5 Hz, H-1a), 4.89 (1H, d, *J* = 13.0 Hz, OC*H*HAr), 4.86 (1H, d, *J* = 13.0 Hz, OCH*H*Ar), 4.83 (1H, ddd, *J*_5,6a_ = 6.8, *J*_4,5_ = 6.3, *J*_5,6b_ = 5.3 Hz, H-5), 4.76 (1H, dd, *J*_6a,6b_ = 11.5, *J*_5,6a_ = 6.8 Hz, H-6a), 4.60 (1H, dd, *J*_6a,6b_ = 11.5, *J*_5,6b_ = 5.3 Hz, H-6b), 4.53 (1H, d, *J*_1a,1b_ = 12.5 Hz, H-1b). ^13^C{^1^H} NMR (126 MHz, CDCl_3_) δ: 166.0, 165.6, 165.1, 164.6, 147.3, 145.0, 133.6, 133.5, 133.3, 133.2, 129.7 (4C), 129.6 (4C), 129.4, 129.0, 128.7, 128.6 (2C), 128.5, 128.43 (2C), 128.36 (2C), 128.32 (2C), 127.7 (2C), 123.6 (2C), 106.9, 76.6, 75.6, 72.0, 63.2, 63.0, 59.6. IR (KBr): 3065, 2963, 1732 cm^−1^. HRMS (ESI) *m*/*z*: [M + Na]^+^ calcd for C_41_H_33_NO_12_Na, 754.1900; found, 754.1902.

#### 3.7.4. Cyclohexyl 1,3,4,6-Tetra-*O*-benzoyl-α-d-tagatofuranoside (**9d**)

According to the general procedure for the glycosidation reaction, compound **9d** (53.2 mg) was obtained from the glycosyl donor **8** (83.5 mg, 0.100 mmol) and cyclohexanol (0.0158 mL, 0.150 mmol) in 78% yield as a 94:6 mixture of α- and β-anomers. Colorless oil. Eluent for column: 10% EtOAc in *n*-hexane for the first column and CHCl_3_ for second column. [α]^23^_D_: +70.1 (*c* 1.00, CHCl_3_). ^1^H NMR (500 MHz, CDCl_3_) δ: 7.96–7.86 (6H, m, Ar*H*), 7.83–7.77 (2H, m, Ar*H*), 7.55–7.43 (4H, m, Ar*H*), 7.39–7.28 (6H, m, Ar*H*), 7.27–7.21 (2H, m, Ar*H*), 6.19 (1H, dd, *J*_4,5_ = 6.5, *J*_3,4_ = 5.2 Hz, H-4), 5.94 (1H, d, *J*_3,4_ = 5.2 Hz, H-3), 4.90 (1H, ddd, *J*_5,6a_ = 6.9, *J*_4,5_ = 6.5, *J*_5,6b_ =5.2 Hz, H-5), 4.77 (1H, d, *J*_1a,1b_ = 12.3 Hz, H-1a), 4.70 (1H, dd, *J*_6a,6b_ = 11.4, *J*_5,6a_ = 6.9 Hz, H-6a), 4.59 (1H, dd, *J*_6a,6b_ = 11.4, *J*_5,6b_ =5.2 Hz, H-6b), 4.57 (1H, d, *J*_1a,1b_ = 12.3 Hz, H-1b), 3.99–3.89 (1H, m, OC*H* of cHex), 1.98–1.85 (1H, m, C*H*_2_ of cHex), 1.78–1.67 (3H, m, C*H*_2_ of cHex), 1.55–1.44 (2H, m, C*H*_2_ of cHex), 1.42–1.32 (2H, m, C*H*_2_ of cHex), 1.29–1.17 (2H, m, C*H*_2_ of cHex). ^13^C{^1^H} NMR (126 MHz, CDCl_3_) δ: 166.1, 165.8, 165.2, 164.7, 133.3 (2C), 133.1, 133.0, 129.7 (2C), 129.63 (2C), 129.61 (2C), 129.59 (2C), 129.52, 129.3, 128.9, 128.7, 128.4 (2C), 128.33 (2C), 128.29 (2C), 128.23 (2C), 106.5, 76.3, 76.1, 72.2, 71.0, 63.3, 60.8, 34.4, 33.7, 25.4, 24.2 (2C). IR (KBr): 3063, 2936, 1728 cm^−1^. HRMS (ESI) *m*/*z*: [M + Na]^+^ calcd for C_40_H_38_O_10_Na, 701.2363; found, 701.2360.

#### 3.7.5. Isopropyl 1,3,4,6-Tetra-*O*-benzoyl-α-d-tagatofuranoside (**9e**)

According to the general procedure of the glycosidation reaction, compound **9e** (48.4 mg) was obtained from the glycosyl donor **8** (83.5 mg, 0.100 mmol) and isopropanol (11.5 μL, 0.150 mmol) in 76% yield as a 93:7 mixture of α- and β-anomers. Colorless oil. Eluent for column: 20% EtOAc in *n*-hexane. *R*_f_ = 0.55 (30% EtOAc in *n*-hexane). [α]^23^_D_ = +79.0 (*c* 1.00, CHCl_3_). ^1^H NMR (500 MHz, CDCl_3_) δ: 7.95–7.89 (6H, m, Ar*H*), 7.83–7.78 (2H, m, Ar*H*), 7.54–7.44 (4H, m, Ar*H*), 7.38–7.29 (6H, m, Ar*H*), 7.27–7.23 (2H, m, Ar*H*), 6.18 (1H, dd, *J*_4,5_ = 6.5, *J*_3,4_ = 5.1 Hz, H-4), 5.92 (1H, d, *J*_3,4_ = 5.1 Hz, H-3), 4.90 (1H, ddd, *J*_5,6a_ = 6.8, *J*_4,5_ = 6.5, *J*_5,6b_ = 5.5 Hz, H-5), 4.81 (1H, d, *J*_1a,1b_ = 12.4 Hz, H-1a), 4.70 (1H, dd, *J*_6a,6b_ = 11.5, *J*_5,6a_ = 6.8 Hz, H-6a), 4.58 (1H, dd, *J*_6a,6b_ = 11.5, *J*_5,6b_ = 5.5 Hz, H-6b), 4.55 (1H, d, *J*_1a,1b_ = 12.4 Hz, H-1b), 4.27 (1H, qq, *J* = 6.1, 6.1 Hz, OC*H*(CH_3_)_2_), 1.29 (3H, d, *J* = 6.1 Hz, OCH(C*H*_3_)_2_), 1.16 (3H, d, *J* = 6.1 Hz, OCH(C*H_3_*)_2_). ^13^C{^1^H} NMR (126 MHz, CDCl_3_) δ: 166.1, 165.8, 165.2, 164.7, 133.4 (2C), 133.1, 133.0, 129.7 (2C), 129.64 (2C), 129.62 (2C), 129.61 (2C), 129.5, 129.3, 128.9, 128.7, 128.5 (2C), 128.4 (2C), 128.31 (2C), 128.25 (2C), 106.6, 76.2, 76.1, 72.2, 65.6, 63.4, 60.6, 24.3, 23.8. IR (film): 3063, 2980, 2932, 1744, 1730, 1603 cm^−1^. HRMS (ESI) *m*/*z*: [M + Na]^+^ calcd for C_37_H_34_O_10_Na, 661.2050; found, 661.2054.

#### 3.7.6. (4-Methoxyphenyl) 1,3,4,6-Tetra-*O*-benzoyl-α-d-tagatofuranoside (**9f**)

According to the general procedure of the glycosidation reaction, compound **9f** (39.1 mg) was obtained from the glycosyl donor (83.5 mg, 0.100 mmol) and 4-methoxyphenol (18.6 mg, 0.150 mmol) in 56% yield as an 89:11 mixture of α- and β-anomers. White solid. Eluent for column: 10% EtOAc in *n*-hexane. *R*_f_ = 0.38 (30% EtOAc in *n*-hexane). Mp 43–45 °C. [α]^19^_D_ = +25.9 (*c* 1.00, CHCl_3_). ^1^H NMR (500 MHz, CDCl_3_) δ: 7.99–7.96 (2H, m, Ar*H*), 7.93–7.89 (2H, m, Ar*H*), 7.87–7.82 (4H, m, Ar*H*), 7.58–7.42 (2H, m, Ar*H*), 7.50–7.44 (2H, m, Ar*H*), 7.43–7.33 (2H, m, Ar*H*), 7.38–7.33 (2H, m, Ar*H*), 7.31–7.24 (4H, m, Ar*H*), 7.14–7.08 (2H, m, Ar*H*), 6.81–6.75 (2H, m, Ar*H*), 6.26 (1H, dd, *J*_3,4_ = 5.3, *J*_4,5_ = 5.3 Hz, H-4), 6.24 (1H, d, *J*_3,4_ = 5.3 Hz, H-3), 5.08 (1H, ddd, *J*_5,6a_ = 7.0, *J*_5,6b_ = 5.5, *J*_4,5_ = 5.3 Hz, H-5), 4.75 (1H, dd, *J*_6a,6b_ = 11.6, *J*_5,6a_ = 7.0 Hz, H-6a), 4.67 (1H, dd, *J*_6a,6b_ = 11.6, *J*_5,6b_ = 5.5 Hz, H-6b), 4.63 (1H, d, *J*_1a,1b_ = 12.3 Hz, H-1a), 4.61 (1H, d, *J*_1a,1b_ = 12.3 Hz, H-1b), 3.75 (3H, s, OC*H*_3_). ^13^C{^1^H} NMR (126 MHz, CDCl_3_) δ: 166.0, 165.5, 165.2, 164.6, 156.5, 145.8, 133.5, 133.4, 133.2, 133.1, 129.72 (2C), 129.66 (2C), 129.63 (4C), 129.5, 129.2, 128.62, 128.58, 128.44 (2C), 128.42 (2C), 128.33 (2C), 128.32 (2C), 123.6 (2C), 114.4 (2C), 108.3, 76.6, 76.2, 72.1, 62.9, 61.0, 55.5. IR (KBr): 3073, 2959, 2928, 2841, 1732, 1603 cm^−1^. HRMS (ESI) *m*/*z*: [M + Na]^+^ calcd for C_41_H_34_O_11_Na, 725.1999; found, 725.1984.

#### 3.7.7. (2-Isopropylphenyl) 1,3,4,6-Tetra-*O*-benzoyl-α-d-tagatofuranoside (**9g**)

According to the general procedure for the glycosidation reaction, compounds **9gα** (10.5 mg) and **9gβ** (4.3 mg) were obtained from the glycosyl donor **8** (83.5 mg, 0.100 mmol) and 2-isopropylphenol (20.4 μL, 0.150 mmol) in 15% and 6% yields, respectively.

**9gα**: Colorless oil. Eluent for column: 8% EtOAc in *n*-hexane. *R*_f_ = 0.44 (30% EtOAc in *n*-hexane). ^1^H NMR (500 MHz, CDCl_3_) δ: 8.00–7.95 (2H, m, Ar*H*), 7.91–7.87 (2H, m, Ar*H*), 7.86–7.80 (4H, m, Ar*H*), 7.57–7.51 (2H, m, Ar*H*), 7.51–7.45 (3H, m, Ar*H*), 7.42–7.36 (2H, m, Ar*H*), 7.33–7.27 (6H, m, Ar*H*), 7.19 (1H, dd, *J* = 7.6, 1.9 Hz, Ar*H*), 7.13 (1H, td, *J* = 7.8, 1.9 Hz, Ar*H*), 7.06 (1H, td, *J* = 7.4, 1.3 Hz, Ar*H*), 6.26 (1H, d, *J*_3,4_ = 5.3 Hz, H-3), 6.25 (1H, dd, *J*_3,4_ = 5.3, *J*_4,5_ = 4.5 Hz, H-4), 5.08 (1H, ddd, *J*_5,6a_ = 7.0, *J*_5,6b_ = 5.4, *J*_4,5_ = 4.5 Hz, H-5), 4.95 (1H, d, *J*_1a,1b_ = 12.3 Hz, H-1a), 4.79 (1H, dd, *J*_6a,6b_ = 11.6, *J*_5,6a_ = 7.0 Hz, H-6a), 4.68 (1H, d, *J*_1a,1b_ = 12.3 Hz, H-1b), 4.66 (1H, dd, *J*_6a,6b_ = 11.6, *J*_5,6b_ = 5.4 Hz, H-6b), 3.39 (1H, qq, *J* = 7.0, 6.8 Hz, -C*H*Me_2_), 1.18 (3H, d, *J* = 6.8 Hz, -C*H*_3_), 0.96 (3H, d, *J* = 7.0 Hz, -C*H*_3_). HRMS (ESI) *m*/*z*: [M + Na]^+^ calcd for C_43_H_38_O_10_Na, 737.2363; found, 737.2365. **9gβ**: Colorless oil. Eluent for column: 12% EtOAc in *n*-hexane. *R*_f_ = 0.39 (30% EtOAc in *n*-hexane). ^1^H NMR (500 MHz, CDCl_3_) δ: 8.02–7.96 (4H, m, Ar*H*), 7.96–7.92 (2H, m, Ar*H*), 7.87–7.82 (2H, m, Ar*H*), 7.75–7.71 (1H, m, Ar*H*), 7.57–7.49 (3H, m, Ar*H*), 7.47–7.37 (3H, m, Ar*H*), 7.35–7.28 (4H, m, Ar*H*), 7.23–7.17 (3H, m, Ar*H*), 7.12 (1H, ddd, *J* = 8.3, 7.3, 2.0 Hz, Ar*H*), 7.05 (1H, td, *J* = 7.5, 1.3 Hz, Ar*H*), 6.28 (1H, dd, *J*_3,4_ = 6.0, *J*_4,5_ = 5.6 Hz, H-4), 6.01 (1H, d, *J*_3,4_ = 6.0 Hz, H-3), 5.04 (1H, dt, *J*_5,6a_ =7.2, *J*_4,5_ = *J*_5,6b_ = 5.6 Hz, H-5), 4.82 (1H, dd, *J*_6a,6b_ = 11.7, *J*_5,6a_ = 7.2 Hz, H-6a), 4.77 (1H, dd, *J*_6a,6b_ = 11.7, *J*_5,6b_ = 5.6 Hz, H-6b), 4.73 (1H, d, *J*_1a,1b_ = 12.1 Hz, H-1a), 4.61 (1H, d, *J*_1a,1b_ = 12.1 Hz, H-1b), 3.45 (1H, qq, *J* = 7.1, 6.8 Hz, -C*H*Me_2_), 1.04 (3H, d, *J* = 6.8 Hz, -C*H*_3_), 0.90 (3H, d, *J* = 7.1 Hz, -C*H*_3_). HRMS (ESI) *m*/*z*: [M + Na]^+^ calcd for C_43_H_38_O_10_Na, 737.2363; found, 737.2371.

#### 3.7.8. (Fmoc-L-Ser-OMe) 1,3,4,6-Tetra-*O*-benzoyl-α-d-tagatofuranoside (**9h**)

According to the general procedure of the glycosidation reaction, compound **9h** (74.4 mg) was obtained from the glycosyl donor **8** (83.5 mg, 0.100 mmol) and Fmoc-L-Ser-OMe (51.2 mg, 0.150 mmol) in 81% yield as a 97:3 mixture of α- and β-anomers. White solid. Eluent for column: 30% EtOAc in *n*-hexane. *R*_f_ = 0.22 (30% EtOAc in *n*-hexane). Mp 64–66 °C. [α]^23^_D_ = +58.6 (*c* 1.00, CHCl_3_). ^1^H NMR (500 MHz, CDCl_3_) δ: 7.95–7.87 (6H, m, Ar*H*), 7.85–7.80 (2H, m, Ar*H*), 7.72 (2H, d, *J* = 7.5 Hz, Ar*H*), 7.61 (2H, d, *J* = 7.5 Hz, Ar*H*), 7.53–7.45 (4H, m, Ar*H*), 7.37–7.25 (12H, m, Ar*H*), 6.13 (1H, dd, *J*_4,5_ = 6.5, *J*_3,4_ = 5.3 Hz, H-4), 5.97 (1H, d, *J*_3,4_ = 5.3 Hz, H-3), 5.82 (1H, d, *J* = 7.9 Hz, N*H*), 4.92 (1H, d, *J*_1a,1b_ =12.5 Hz, H-1a), 4.87 (1H, ddd, *J*_5,6a_ = 7.2, *J*_4,5_ = 6.5, *J*_5,6b_ = 5.2 Hz, H-5), 4.70 (1H, dd, *J*_6a,6b_ = 11.6, *J*_5,6a_ = 7.2 Hz, H-6a), 4.62 (1H, dt, *J* = 7.9, 3.6 Hz, NHC*H*), 4.50 (1H, dd, *J*_6a,6b_ = 11.6, *J*_5,6b_ = 5.3 Hz, H-6b), 4.48 (1H, d, *J*_1a,1b_ = 12.5 Hz, H-1b), 4.38 (1H, dd, *J* = 10.5, 7.2 Hz, ArCHC*H*H), 4.33 (1H, dd, *J* = 10.5, 7.2 Hz, ArCHCH*H*), 4.22 (1H, t, *J* = 7.2 Hz, ArC*H*), 4.13 (1H, dd, *J* = 9.8, 3.6 Hz, NHCH_2_C*H*H), 4.09 (1H, dd, *J* = 9.8, 3.6 Hz, NHCH_2_CH*H*), 3.57 (3H, s). ^13^C{^1^H} NMR (126 MHz, CDCl_3_) δ: 170.2, 166.0, 165.6, 165.0, 164.5, 155.8, 143.8, 143.7, 141.18, 141.16, 133.52, 133.47, 133.23, 133.01, 129.69 (2C), 129.66 (2C), 129.63 (2C), 129.59 (2C), 129.3, 129.1, 128.7 (2C), 128.5 (2C), 128.4 (2C), 128.3 (2C), 128.2 (2C), 127.6 (2C), 127.04, 127.00, 125.2, 125.1, 119.9, 106.4, 76.6, 75.3, 71.9, 67.4, 63.1 62.3, 59.7, 54.0, 52.6 (2C), 47.0. IR (KBr): 3067, 2959, 1724, 1601 cm^−1^. HRMS (ESI) *m*/*z*: [M + Na]^+^ calcd for C_53_H_45_NO_14_Na, 942.2738; found, 942.2735.

#### 3.7.9. Methyl 5-*O*-(1,3,4,6-tetra-*O*-benzoyl-α-d-tagatofuranosyl)-2,3-*O*-isopropylidene-β-d-ribofuranoside (**9i**)

According to the general procedure of the glycosidation reaction, compound **9i** (64.6 mg) was obtained from the glycosyl donor **8** (83.5 mg, 0.100 mmol) and methyl 2,3-*O*-isopropylidene-β-d-ribofuranoside (26.2 μL, 0.150 mmol) in 82% yield as an 88:12 mixture of α- and β-anomers. White solid. Eluent for column: 15% EtOAc in *n*-hexane. *R*_f_ = 0.29 (30% EtOAc in *n*-hexane). Mp 45–47 °C. [α]^21^_D_ = +36.2 (*c* 1.00, CHCl_3_). ^1^H NMR (500 MHz, CDCl_3_) δ: 7.98–7.92 (6H, m, Ar*H*), 7.84–7.77 (2H, m, Ar*H*), 7.56–7.49 (3H, m, Ar*H*), 7.48–7.44 (1H, m, Ar*H*), 7.39–7.32 (6H, m, Ar*H*), 7.28–7.22 (2H, m, Ar*H*), 6.18 (1H, dd, *J*_4,5_ = 6.5, *J*_3,4_ = 5.3 Hz, H-4), 5.98 (1H, d, *J*_3,4_ = 5.3 Hz, H-3), 4.95 (1H, s, H-1′), 4.93 (1H, d, *J*_1a,1b_ = 12.4 Hz, H-1a), 4.92 (1H, ddd, *J*_5,6a_ = 6.8, *J*_4,5_ = 6.5, *J*_5,6b_ = 5.4 Hz, H-5), 4.73 (1H, dd, *J*_6a,6b_ = 11.6, *J*_5,6a_ = 6.8 Hz, H-6a), 4.64 (1H, dd, *J*_2′,3′_ = 6.0, *J*_3′,4′_ = 1.0 Hz, H-3′), 4.57 (1H, dd, *J*_6a,6b_ = 11.6, *J*_5,6a_ = 5.4 Hz, H-6b), 4.51 (1H, d, *J*_1a,1b_ = 12.4 Hz, H-1b), 4.49 (1H, d, *J*_2′,3′_ = 6.0 Hz, H-2′), 4.34 (1H, ddd, *J*_4′,5b′_ = 9.3, *J*_4′,5a′_ = 5.7, *J*_3′,4′_ = 1.0 Hz, H-4′), 3.79 (1H, dd, *J*_5a′,5b′_ = 9.3, *J*_4′,5a′_ = 5.7 Hz, H-5a′), 3.61 (1H, t, *J*_5a′,5b′_ = 9.3, *J*_4′,5a′_ = 9.3 Hz, H-5b), 3.33 (3H, s, OC*H*_3_), 1.46 (3H, s, CC*H*_3_), 1.18 (3H, s, CC*H_3_*). ^13^C{^1^H} NMR (126 MHz, CDCl_3_) δ: 166.0, 165.6, 165.1, 164.6, 133.5, 133.4, 133.2, 133.1, 129.69 (2C), 129.66 (2C), 129.64 (2C), 129.63 (2C), 129.5, 129.2, 128.8, 128.60, 128.5 (2C), 128.37 (2C), 128.35 (2C), 128.26 (2C), 112.3, 109.4, 106.4, 85.0, 84.9, 82.0, 76.4, 75.5, 72.0, 63.1, 62.7, 59.5, 55.0, 26.3, 24.6. IR (KBr): 3065, 3032, 2988, 2940, 1736, 1719, 1601 cm^−1^. HRMS (ESI) *m*/*z*: [M + Na]^+^ calcd for C_43_H_42_O_14_Na, 805.2472; found, 805.2470.

#### 3.7.10. Methyl 6-*O*-(1,3,4,6-tetra-*O*-benzoyl-α-d-togatofuranosyl)-2,3,4-tri-*O*-benzyl-α-d-glucopyranoside (**9j**)

According to the general procedure of the glycosidation reaction, compound **9j** (81.7 mg) was obtained from the glycosyl donor **8** (83.5 mg, 0.100 mmol) and methyl 2,3,4-tri-*O*-benzyl-α-d-glucopyranoside (69.6 mg, 0.150 mmol) in 78% yield as a 96:4 mixture of α- and β-anomers. Colorless oil. Eluent for column: 15% EtOAc in *n*-hexane. *R*_f_ = 0.31 (30% EtOAc in *n*-hexane). [α]^22^_D_ = +65.5 (*c* 1.00, CHCl_3_). ^1^H NMR (500 MHz, CDCl_3_) δ: 7.98–7.93 (4H, m), 7.93–7.89 (2H, m), 7.82–7.74 (2H, m), 7.55–7.42 (4H, m), 7.38–7.20 (23H, m), 6.15 (1H, dd, *J*_4,5_ = 6.7, *J*_3,4_ = 5.3 Hz, H-4), 5.92 (1H, d, *J*_3,4_ = 5.3 Hz, H-3), 4.96 (1H, d, *J* = 10.9 Hz), 4.91 (1H, d, *J* = 11.0 Hz), 4.85 (1H, d, *J* = 12.0 Hz), 4.82 (1H, ddd, *J*_5,6a_ = 6.8, *J*_4,5_ = 6.7, *J*_5,6b_ = 6.0 Hz, H-5), 4.80 (1H, d, *J* = 10.9 Hz), 4.66 (1H, dd, *J*_6a,6b_ = 11.6, *J*_5,6a_ = 6.8 Hz, H-6a), 4.64 (1H, d, *J* = 11.0 Hz), 4.61 (1H, d, *J* = 12.0 Hz), 4.54 (1H, dd, *J*_6a,6b_ = 11.6, *J*_5,6b_ = 6.0 Hz, H-6b), 4.54 (1H, d, *J* = 12.0 Hz), 4.49 (1H, d, *J*_1′,2′_ = 3.7 Hz, H-1′), 4.47 (1H, d, *J* = 12.0 Hz), 3.97 (1H, t, *J*_2′,3′_ = 9.2, *J*_3′,4′_ = 9.2 Hz, H-3′), 3.90–3.84 (1H, m, H-6a), 3.81–3.74 (2H, m, H-5, 6b), 3.44 (1H, t, *J*_3′,4′_ = 9.2, *J*_4′,5′_ = 9.2 Hz, H-4′), 3.37 (1H, dd, *J*_2′,3′_ = 9.2, *J*_1′,2′_ = 3.7 Hz, H-2′), 3.37 (3H, s). ^13^C{^1^H} NMR (126 MHz, CDCl_3_) δ: 166.0, 165.6, 165.1, 164.7, 138.7, 138.1, 138.0, 133.4, 133.3, 133.04, 133.01, 129.7 (2C), 129.63 (2C), 129.61 (2C), 129.59 (2C), 129.5, 129.4, 128.9, 128.6, 128.5 (2C), 128.39 (2C), 128.36 (2C), 128.31 (4C), 128.29 (2C), 128.2 (2C), 127.91 (2C), 127.87 (4C), 127.7 (2C), 127.5, 106.2, 97.5, 82.0, 80.1, 78.0, 75.9, 75.6, 75.4, 75.0, 73.1, 72.0, 69.4, 63.1, 61.4, 60.0, 55.1. IR (film): 3065, 3032, 2936, 1736, 1601 cm^−1^. HRMS (ESI) *m*/*z*: [M + Na]^+^ calcd for C_62_H_58_O_15_Na, 1065.3673; found, 1065.3670.

### 3.8. 3-O-Benzoyl-1-O-(1,3,4,6-tetra-O-benzoyl-α-d-tagatofuranosyl)-N-stearoyl-d-erythro-sphingosine (***10***)

According to the general procedure for the glycosidation reaction in the presence of activated molecular sieves 4 Å (100 mg), compound **10** (114 mg) was obtained from the glycosyl donor **8** (100 mg, 0.12 mmol) and ceramide **2** (67.0 mg, 0.100 mmol) in 91% yield as a white solid. Eluent for column: 17% EtOAc in *n*-hexane. *R*_f_ = 0.62 (40% EtOAc in *n*-hexane). Mp 76–78 °C. ^1^H NMR (500 MHz, CDCl_3_) δ: 8.01–7.90 (6H, m, Ar*H*), 7.85–7.78 (4H, m, Ar*H*), 7.56–7.43 (5H, m, Ar*H*), 7.43–7.33 (6H, m, Ar*H*), 7.31–7.23 (4H, m, Ar*H*), 6.12 (1H, dd, *J*_4,5_ = 6.5, *J*_3,4_ = 5.2 Hz, H-4), 5.96 (1H, d, *J*_3,4_ = 5.2 Hz, H-3), 5.86 (1H, dt, *J*_4′,5′_ = 15.2, *J*_5′,6′_ = 7.1 Hz, H-5′), 5.80 (1H, d, *J*_2′,NH_ = 8.9 Hz, NH), 5.58 (1H, dd, *J*_2′,3′_ = 7.2, *J*_3′,4′_ = 7.2 Hz, H-3′), 5.49 (1H, ddt, *J*_4′,5′_ = 15.2, *J*_3′,4′_ = 7.2, *J*_4′,6′_ = 1.4 Hz, H-4′), 4.99 (1H, d, *J*_1a,1b_ = 12.4 Hz, H-1a), 4.85 (1H, ddd, *J*_5,6a_ = 6.8, *J*_4,5_ = 6.5, *J_5,_*_6b_ = 5.4 Hz, H-5), 4.70 (1H, dd, *J*_6a,6b_ = 11.6, *J*_5,6a_ = 6.8 Hz, H-6a), 4.54 (1H, dd, *J*_6a,6b_ = 11.6, *J_5,_*_6b_ = 5.4 Hz, H-6b), 4.58–4.51 (1H, m, H-2′), 4.42 (1H, d, *J*_1a,1b_ = 12.4 Hz, H-1b), 3.93 (2H, m, H-1′), 2.06 (2H, t, *J* = 7.7 Hz, CH_2_), 2.02–1.96 (2H, m, CH_2_), 1.61–1.47 (2H, m, CH_2_), 1.35–1.15 (50H, m, CH_2_), 0.88 (3H, t, *J* = 7.0, CH_3_), 0.87 (3H, t, *J* = 7.0, CH_3_). ^13^C{^1^H} NMR (126 MHz, CDCl_3_) δ: 173.0, 166.2, 166.1, 165.5, 165.2, 164.8, 137.4, 133.7, 133.6, 133.4, 133.3, 133.1, 130.2, 129.88 (2C), 129.86 (3C), 129.82 (2C), 129.80, 129.78 (2C), 129.6, 129.2, 128.9, 128.74 (2C), 128.72, 128.6 (2C), 128.53 (2C), 128.47 (2C), 128.4 (2C), 124.7, 106.8, 76.8, 75.4, 74.9, 72.1, 63.2, 60.7, 59.9, 51.3, 36.9, 32.5, 32.1 (2C), 29.9 (4C), 29.84 (3C), 29.81 (3C), 29.77 (2C), 29.7, 29.6, 29.54, 29.51 (3C), 29.44, 29.39, 29.0, 25.9, 22.8 (2C), 14.3 (2C). IR (KBr): 3385, 2920, 2851, 1719, 1655 cm^−1^.

### 3.9. 1-O-(α-d-Tagatofuranosyl)-N-stearoyl-d-erythro-sphingosine (***11***)

To a solution of glycoside **10** (80.0 mg, 64.1 μmol) in a 1:1 mixture of MeOH/CHCl_3_ (2 mL), a solution of 5 M NaOMe in MeOH (0.032 mL, 0.16 mmol) was added at room temperature. After 5 h, an excess of ion exchange resin (Amberlite FPC3500) was introduced into the reaction mixture, and the resulting suspension was filtered through a Celite pad. After removal of the solvent, the residue was purified by column chromatography on silica gel (7%MeOH in CHCl_3_), yielding the desired cerebroside **11** (35.3 mg) in 76% yield as a white solid. *R*_f_ = 0.31 (10% MeOH in CHCl_3_). Mp 118–120 °C. ^1^H NMR (500 MHz, CDCl_3_/CD_3_OD = 1:1) δ: 5.72 (1H, dt, *J*_4′,5′_ = 15.3, *J*_5′,6′_ = 6.8 Hz, H-5′), 5.45 (1H, dd, *J*_4′,5′_ = 15.3, *J*_3′,4′_ = 7.2 Hz, H-4′), 4.54 (1H, t, *J* = 5.8 Hz), 4.13–4.11 (1H, m), 4.08 (1H, t, *J* = 7.1 Hz), 4.02 (1H, d, *J* = 5.1 Hz), 3.96–3.93 (1H, m), 3.81–3.68 (5H, m), 3.60 (1H, dd, *J* = 10.1, 3.5 Hz), 2.19 (2H, t, *J* = 7.6 Hz, CH_2_), 2.06–2.00 (2H, m, CH_2_), 1.64–1.56 (2H, m, CH_2_), 1.43–1.18 (50H, m, CH_2_), 0.89 (6H, t, *J* = 6.9 Hz, CH_3_). ^13^C{^1^H} NMR (126 MHz, CDCl_3_/CD_3_OD = 1:1) δ: 175.4, 134.7, 129.7, 108.6, 80.6, 78.3, 75.2, 72.6, 72.1, 61.2, 60.8, 58.9, 54.3, 36.9, 32.9, 32.5 (2C), 30.25, 30.22 (8C), 30.19 (2C), 30.17, 30.1 (2C), 30.0, 29.88, 29.87 (2C), 29.85, 29.8, 26.5, 23.2 (2C), 14.3 (2C). IR (KBr): 3294 (br), 2918, 2851, 1638 cm^−1^.

### 3.10. 3-O-Benzoyl-1-O-(1,3,4,6-tetra-O-benzoyl-α-d-tagatofuranosyl)-N-methyl-N-stearoyl-d-erythro-sphingosine (***13***)

According to the general procedure for the glycosidation reaction (**4.6**), compound **13** (114 mg) was obtained from the glycosyl donor **8** (100 mg, 0.12 mmol) and *N*-methylceramide **12** (67.0 mg, 0.0979 mmol) in 92% yield as a colorless syrup. ^1^H NMR (500 MHz, CDCl_3_, 4:1 mixture of rotamers) δ: 8.06–7.77 (10H, m, Ar*H*), 7.56–7.38 (5H, m, Ar*H*), 7.38–7.22 (10H, m, Ar*H*), 6.10 (0.8H, dd, *J*_4,5_ = 6.4, *J*_3,4_ = 5.1 Hz, major-H-4), 6.10–6.06 (0.2H, m, minor-H-4), 5.92 (1H, d, *J*_3,4_ = 5.1 Hz, H-3), 5.91–5.86 (1H, m, H-5′), 5.67–5.57 (1H, m, H-3′), 5.46 (1H, dd, *J*_4′,5′_ =15.4, *J*_3′,4′_ = 7.5 Hz, H-4′), 5.16 (0.8H, br s, major-H-2′), 4.95 (0.8H, ddd, *J*_5,6a_ = 7.1, *J*_4,5_ = 6.4, *J_5,_*_6b_ = 5.2 Hz, major-H-5), 4.87 (1H, d, *J*_1a,1b_ = 12.3 Hz, H-1a), 4.84–4.78 (0.2H, m, minor-H-5), 4.70 (0.8H, dd, *J*_6a,6b_ = 11.6, *J*_5,6a_ = 7.1 Hz, major-H-6a), 4.67–4.61 (0.2H, m, minor-H-6a), 4.55 (1H, dd, *J*_6a,6b_ = 11.6, *J_5,_*_6b_ = 5.2 Hz, H-6b), 4.53–4.46 (1H, m, H-6b), 4.49 (0.2H, d, *J*_1a,1b_ = 12.3 Hz, minor-H-1b), 4.42 (0.8H, d, *J*_1a,1b_ = 12.3 Hz, major-H-1b), 4.35–4.28 (0.2H, m, minor-H-2′), 4.06 (0.8H, dd, *J*_1′a,1′b_ = 10.6, *J*_1′a,2′_ = 3.8 Hz, major-H-1′a), 3.99 (0.2H, d, *J*_1′a,1′b_ = 9.8, *J*_1′a, 2′_ = 4.1 Hz, minor-H-1′a), 3.96–3.85 (1H, m, H-1′b), 2.89 (2.4H, s, major-N-CH_3_), 2.79 (0.6H, s, minor-N-CH_3_), 2.46–2.38 (0.4H, m, minor-CH_2_), 2.33–2.19 (1.6H, m, major-CH_2_), 2.06–1.91 (2H, m, CH_2_), 1.68–1.55 (2H, m, CH_2_), 1.37–1.16 (50H, m, CH_2_), 0.88 (6H, t, *J* = 6.8, CH_3_). ^13^C{^1^H} NMR (126 MHz, CDCl_3_) δ: 174.5, 166.2, 165.7, 165.5, 165.1, 164.7, 137.9, 133.6, 133.5, 133.4, 133.2, 133.1, 130.2, 129.82 (2C), 129.80 (2C), 129.76 (5C), 129.7 (2C), 129.3, 128.9, 128.8, 128.7, 128.6 (2C), 128.53 (2C), 128.49 (2C), 128.41 (2C), 128.39 (2C), 125.4, 106.6, 76.7, 75.8, 74.1, 72.2, 63.3, 60.3, 59.5, 34.1, 32.3, 32.0 (2C), 29.84 (5C), 29.81 (3C), 29.78 (3C), 29.76, 29.73 (2C), 29.68, 29.66, 29.6, 29.5 (3C), 29.3, 29.0 25.2, 22.8 (2C), 14.2 (2C). IR (KBr): 2924, 2853, 1728, 1651, 1603 cm^−1^. HRMS (ESI) *m*/*z*: [M + Na]^+^ calcd for C_78_H_103_NO_13_Na, 1284.7327; found, 1284.7324.

### 3.11. 1-O-(α-d-Tagatofuranosyl)-N-methyl-N-stearoyl-d-erythro-sphingosine (***14***)

Cerebroside **14** (51.7 mg) was obtained from glycoside **13** (93.0 mg, 0.0737 mmol) in a manner similar to that described for the synthesis of cerebroside **11** in 95% yield as a white solid. Eluent for column: 5% MeOH in CHCl_3_. *R*_f_ = 0.31 (10% MeOH in CHCl_3_). Mp 58–60 °C. ^1^H NMR (400 MHz, CDCl_3_/CD_3_OD = 1:1, 2:1 mixture of rotamers) δ: 5.81–5.63 (1H, m, H-5′), 5.40 (1H, ddd, *J* = 15.4, 7.9, 3.3 Hz, H-4′), 4.48 (2/3H, t, *J* = 5.7 Hz), 4.44 (1/3H, t, *J* = 5.7 Hz), 4.27–4.10 (2H, m), 4.03 (1/3H, d, *J* = 5.1 Hz), 4.00 (2/3H, d, *J* = 5.1 Hz), 3.93–3.61 (7H, m), 2.96 (2H, s, N-CH_3_), 2.82 (1H, s, N-CH_3_), 2.46–2.23 (2H, m), 2.14–1.91 (2H, m), 1.67–1.51 (2H, m), 1.49–1.15 (50H, m), 0.89 (6H, t, *J* = 6.7 Hz, CH_3_). ^13^C{^1^H} NMR (100 MHz, CDCl_3_/CD_3_OD = 1:1) δ: 175.8, 135.0, 130.4, 108.7, 80.7, 78.4, 75.6, 72.2, 71.4, 60.9, 60.3, 58.9, 34.6, 32.9, 32.5 (2C), 30.3 (13C), 30.23 (2C), 30.18 (2C), 30.1, 29.94 (2C), 29.88, 29.7, 25.8, 23.2 (2C), 14.4 (2C). IR (KBr): 3363 (br), 2913, 2849, 1616 cm^−1^. HRMS (ESI) *m*/*z*: [M + Na]^+^ calcd for C_43_H_83_NO_8_Na, 764.6016; found, 764.6020.

## 4. Conclusions

In conclusion, we demonstrated the utility of a benzoyl-protected d-tagatofuranosyl donor. This donor predominantly yielded α-glycosides in d-tagatofuranosidation with various glycosyl acceptors, similar to the 3,4-*O*-isopropylidene-protected tagatofuranosyl donor. The synthesis of α-d-tagatofuranosylceramide has been challenging because of the deprotection issue associated with the 3,4-*O*-isopropylidene-protected tagatofuranosyl donor. However, in this method, the benzoyl protecting groups can be easily removed after the glycosidation reaction. We successfully synthesized α-d-tagatofuranosylceramide for the first time in this study. This strategy may serve as a valuable method for the synthesis of new d-tagatose derivatives.

## Figures and Tables

**Table 1:** Glycosidation of d-tagatofuranosyl donor **8**.

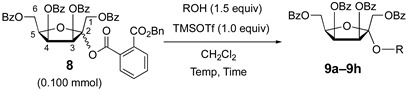						
Entry	R	9	Temp (°C)	Time (min)	Yield (%)	α/β *^a^*
1	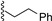	**9a**	−20	30	80	99:1
2	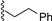	**9a**	rt	30	69	85:15
3	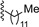	**9b**	0	30	72	99:1
4	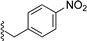	**9c**	−20 to −10	60	81	92:8
5	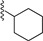	**9d**	−20 to −10	60	78	94:6
6	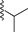	**9e**	−20 to −10	60	76	93:7
7 *^b^*	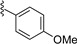	**9f**	−20 to 0	60	56	89:11
8 *^b^*	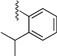	**9g**	−20 to 0	60	15 (α) + 6 (β)	71:29 *^c^*
9	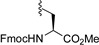	**9h**	−20	30	81	97:3
10	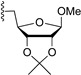	**9i**	−20	30	82	88:12
11	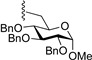	**9j**	−20	30	78	96:4

*^a^* The ratio was determined by 1H NMR except for entry 8. *^b^* An additional TMSOTf (1 equiv.) was added after 20 min. *^c^* Based on isolated yields.

## Data Availability

The original contributions presented in this study are included in the article/Supplementary Materials. Further inquiries can be directed to the corresponding author.

## Supplementary Materials

The following supporting information can be downloaded at: https://www.mdpi.com/article/10.3390/ijms26178459/s1.

## Abbreviations

The following abbreviations are used in this manuscript:

Bz	Benzoyl
DMAP	Benzyl
TBAF	Tetrabutylammonium fluoride
TBDPS	*tert*-Butyldiphenylsilyl
NBS	*N*-Bromosuccinimide
Bn	Benzyl
DCC	*N*,*N’*-Dicyclohexylcarbodiimide
Fmoc	9-Fluorenylmethyloxycarbonyl
TMSOTf	Trimethylsilyl trifluoromethanesulfonate
