# Supplementary material for: α-Selective Glycosidation of the Rare Sugar d-Tagatofuranose and the Synthesis of α-d-Tagatofuranosylceramide
Source: Int J Mol Sci. 2025 Aug 30;26(17):8459. doi: 10.3390/ijms26178459 (PMC12429656; doi:10.3390/ijms26178459)

Supplementary Materials

α-Selective glycosidation of the rare sugar D-tagatofuranose and the synthesis of α-D-tagatofuranosylceramide

Yui Makura [1], Akihiro Iyoshi[a], Makito Horiuchi [2], Yiming Hu [1], Masakazu Tanaka [1], and Atsushi Ueda [1,*]

[1] *Graduate School of Biomedical Sciences, Nagasaki University, 1-14 Bunkyo-machi, Nagasaki 852-8521, Japan*

[2] *School of Pharmaceutical Sciences, Nagasaki University, 1-14 Bunkyo-machi, Nagasaki 852-8521, Japan*

E-mail for A. U.: aueda@nagasaki-u.ac.jp

Table of Contents

1. NMR spectra ··· S2

1. NMR spectra.

^{1}H NMR of **3** (500 MHz, $CDCl_3$)

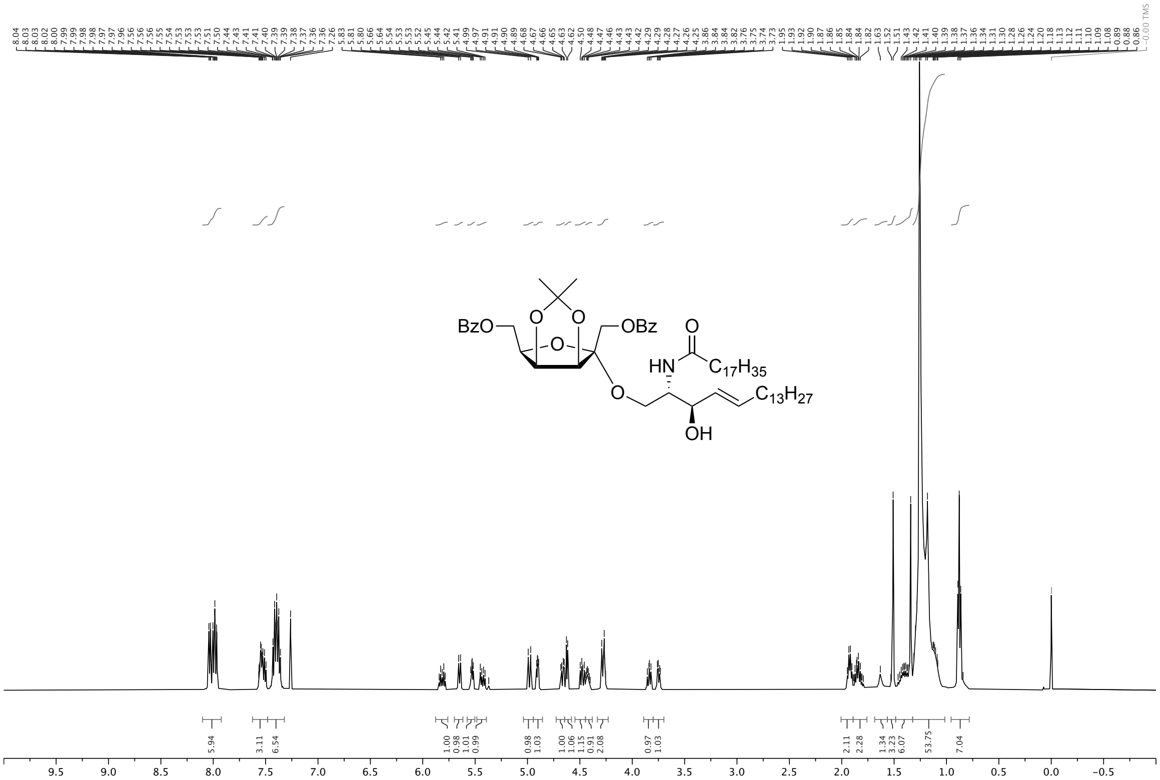

^{13}C NMR of **3** (126 MHz, $CDCl_3$)

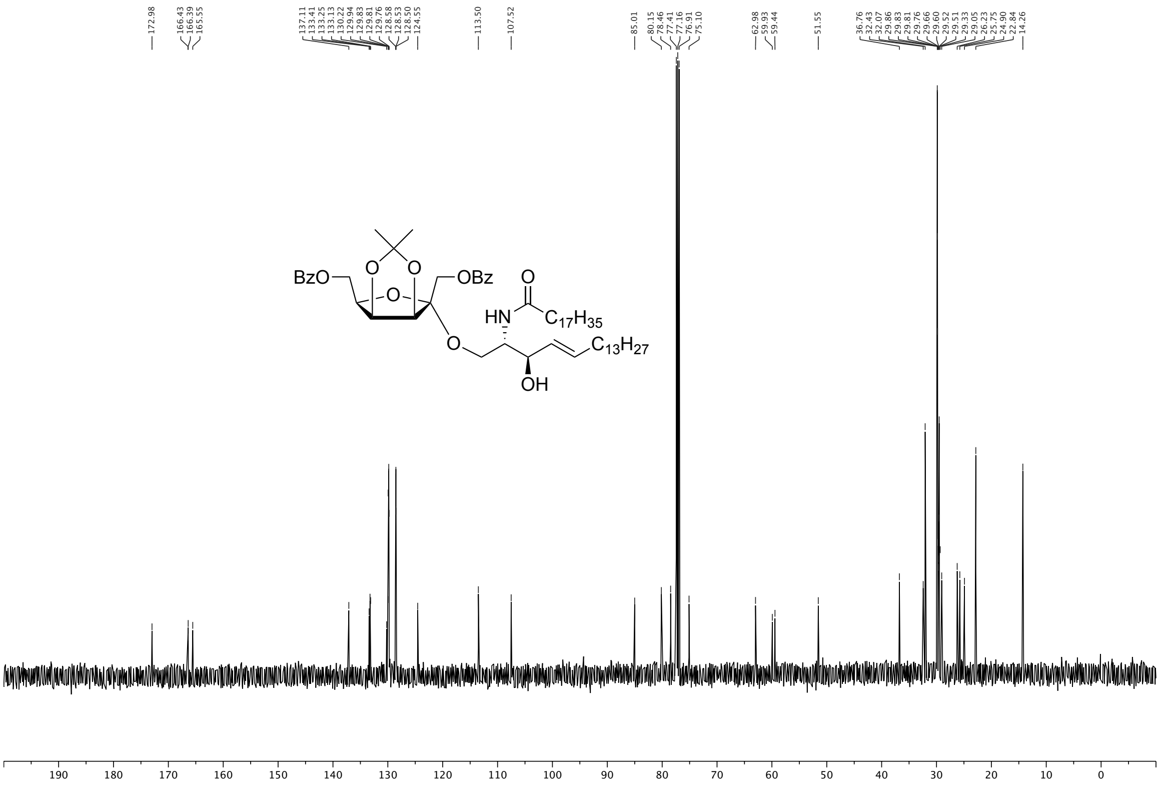

^{1}H NMR of **5** (400 MHz, $CDCl_3$)

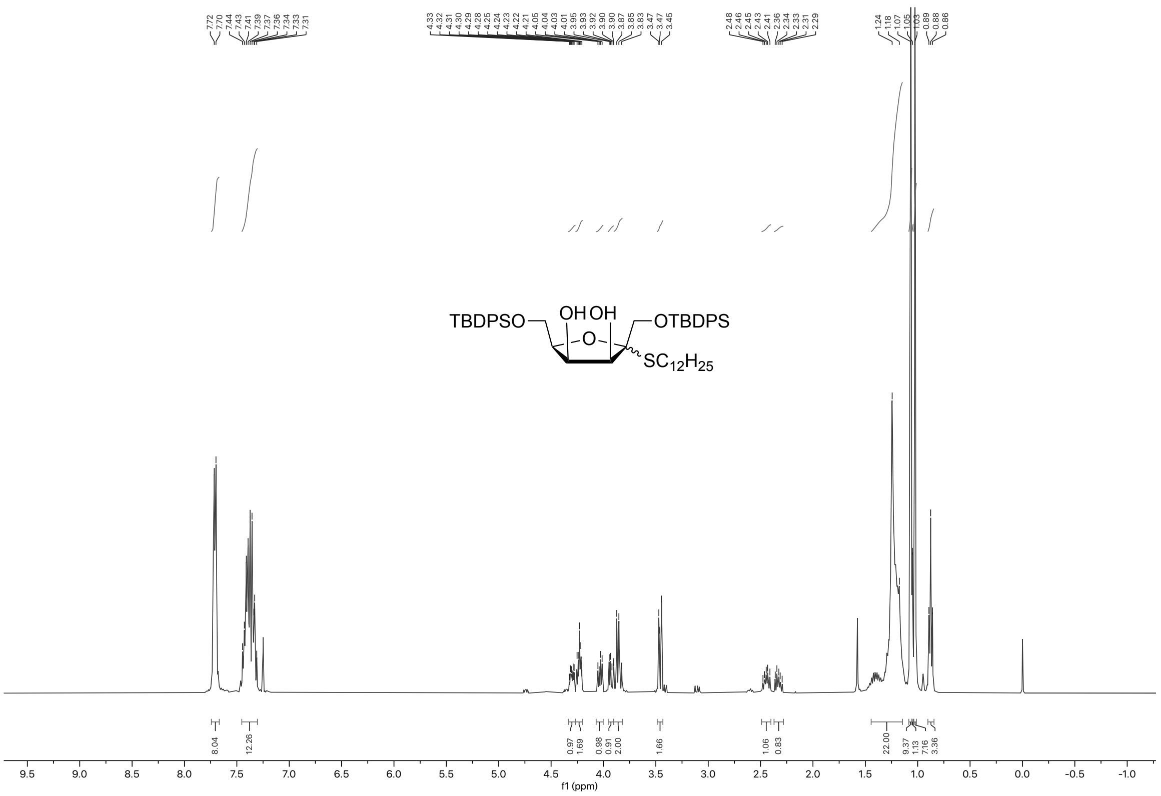

^{13}C NMR of **5** (100 MHz, $CDCl_3$)

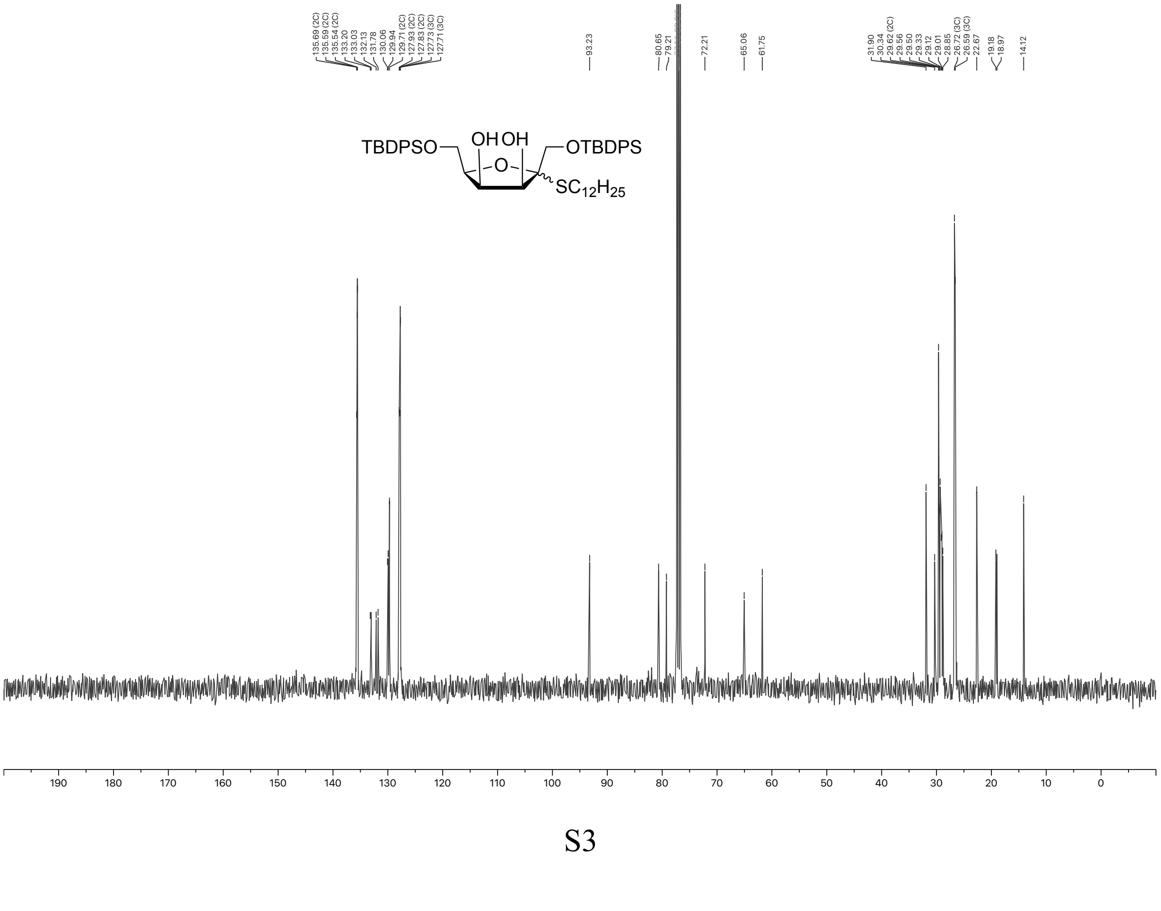

^{1}H NMR of **6** (400 MHz, $CDCl_3$)

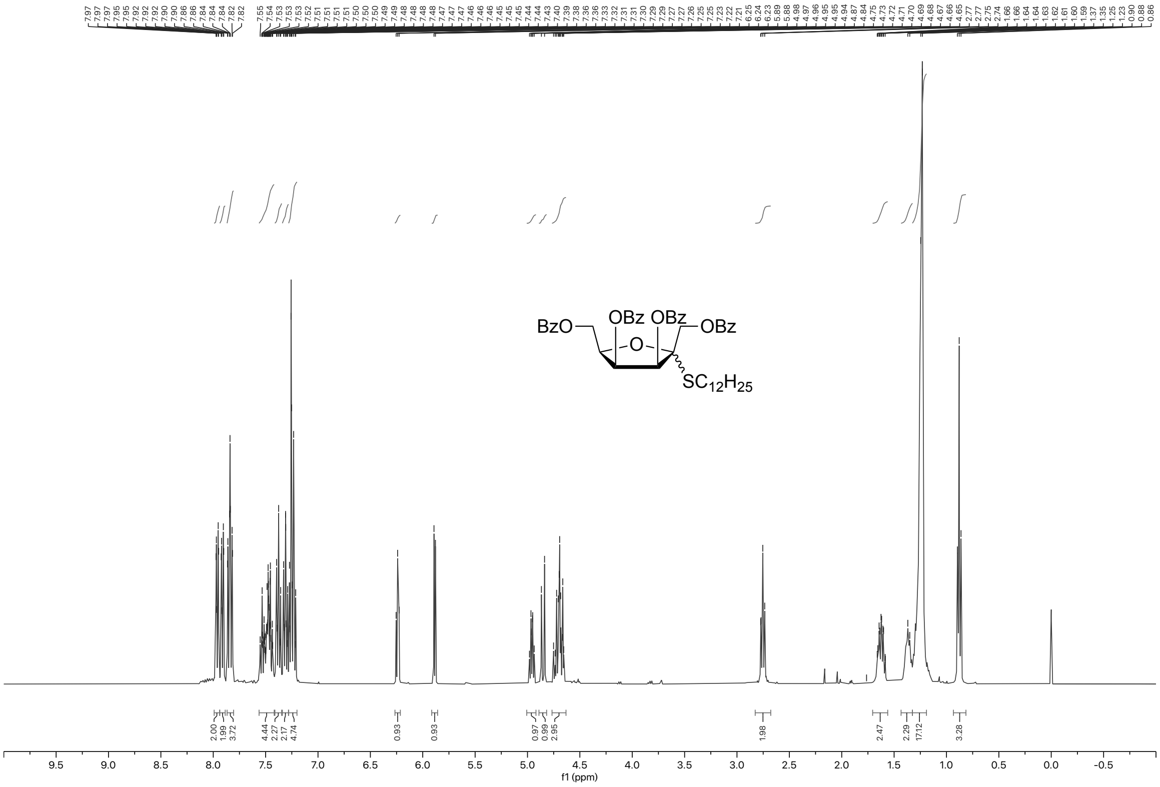

^{13}C NMR of **6** (100 MHz, $CDCl_3$)

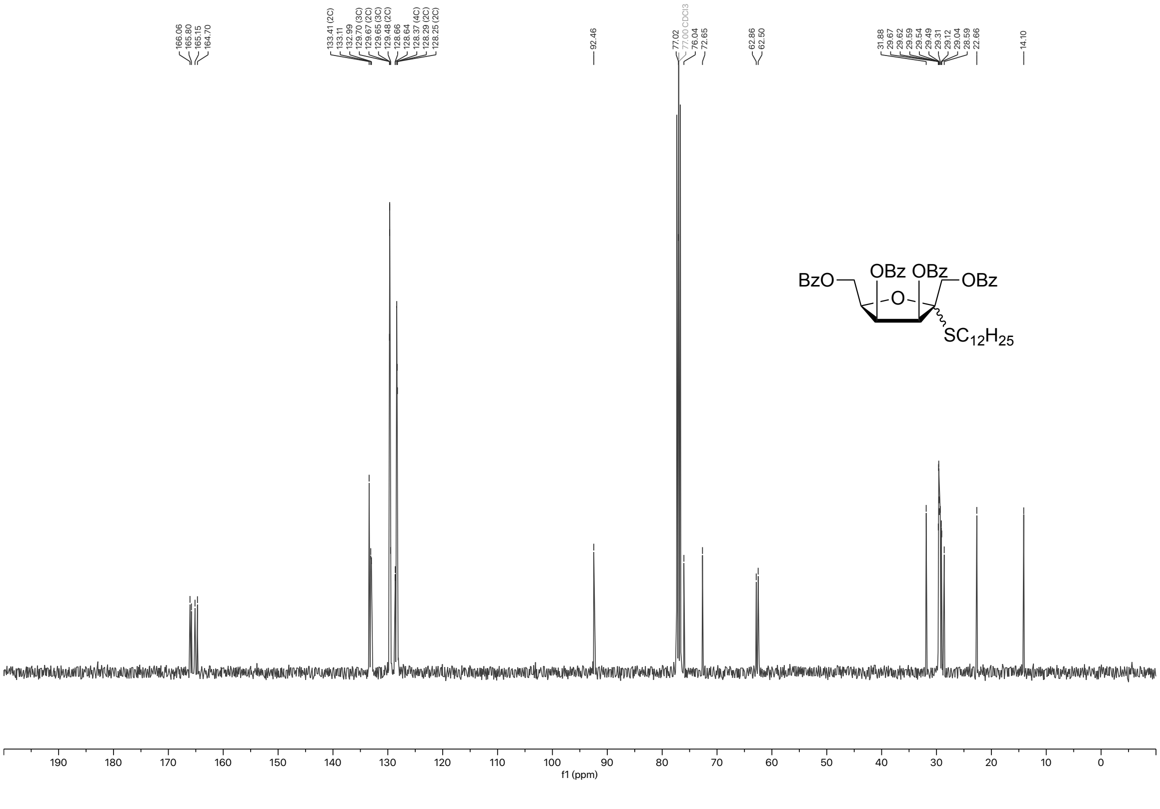

^{1}H NMR of **7** (400 MHz, $CDCl_3$)

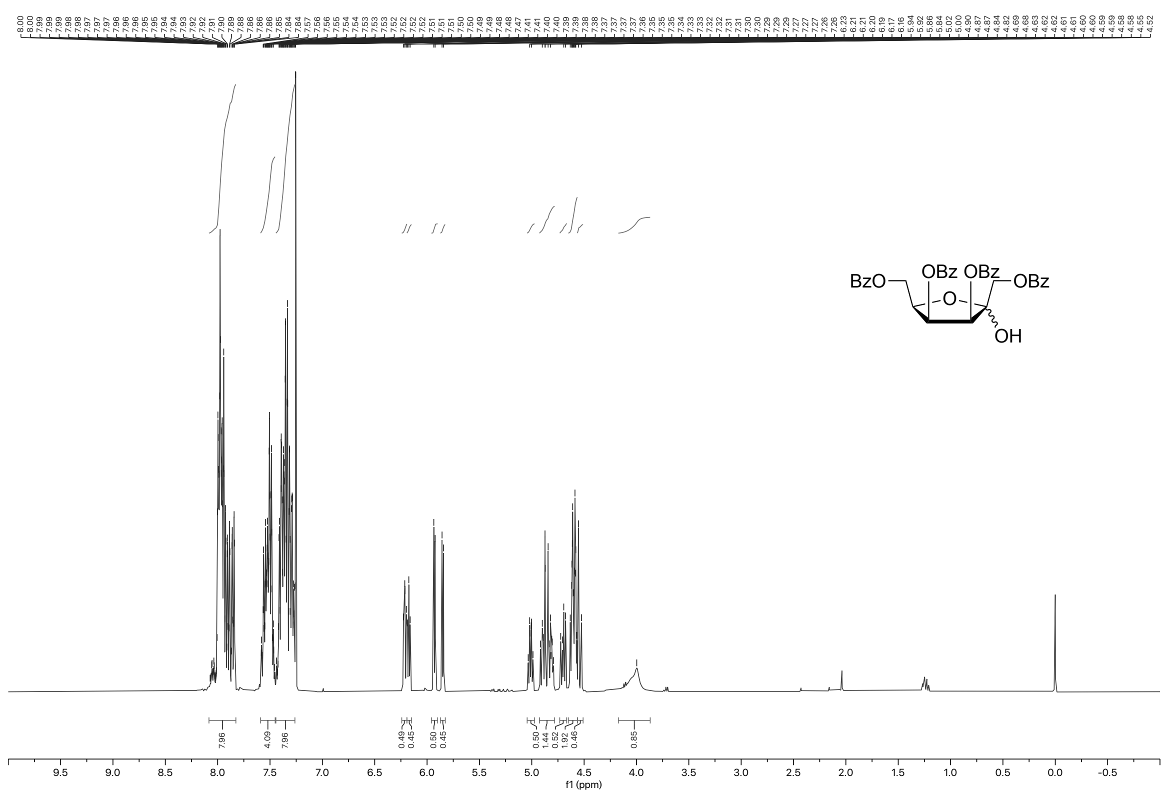

^{13}C NMR of **7** (100 MHz, $CDCl_3$)

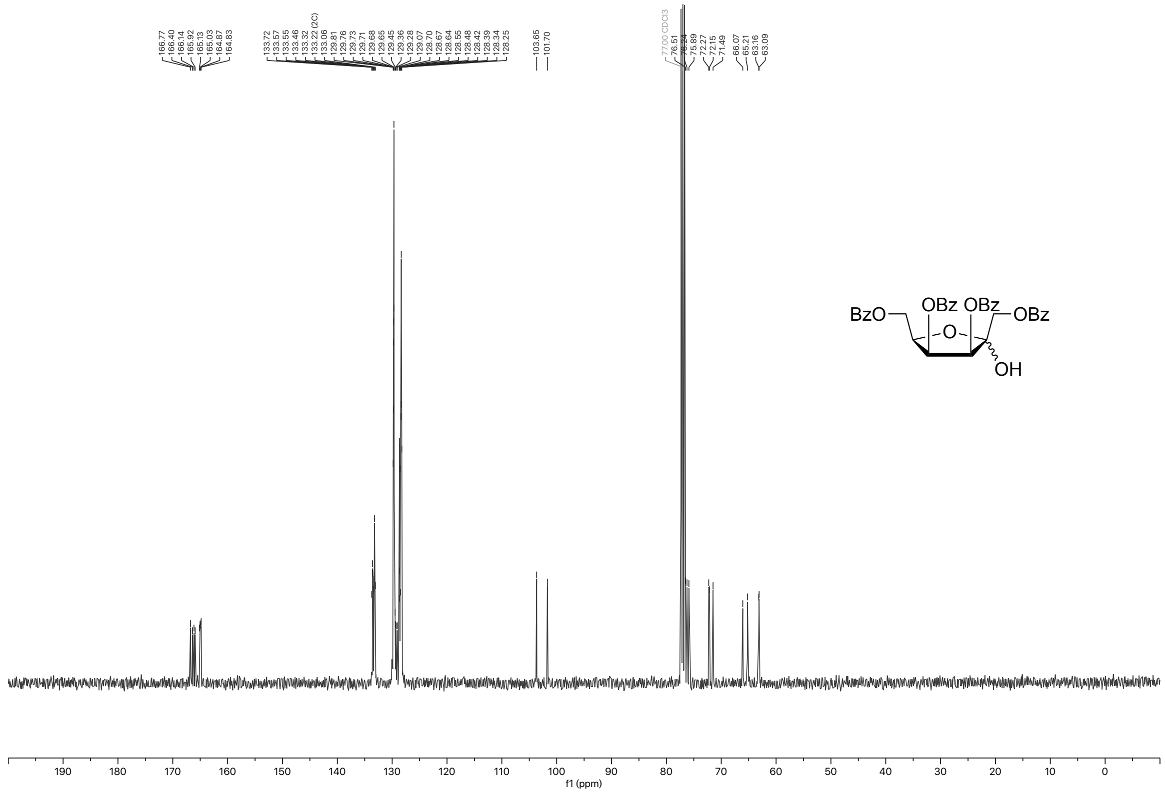

^{1}H NMR of **8** (400 MHz, $CDCl_3$)

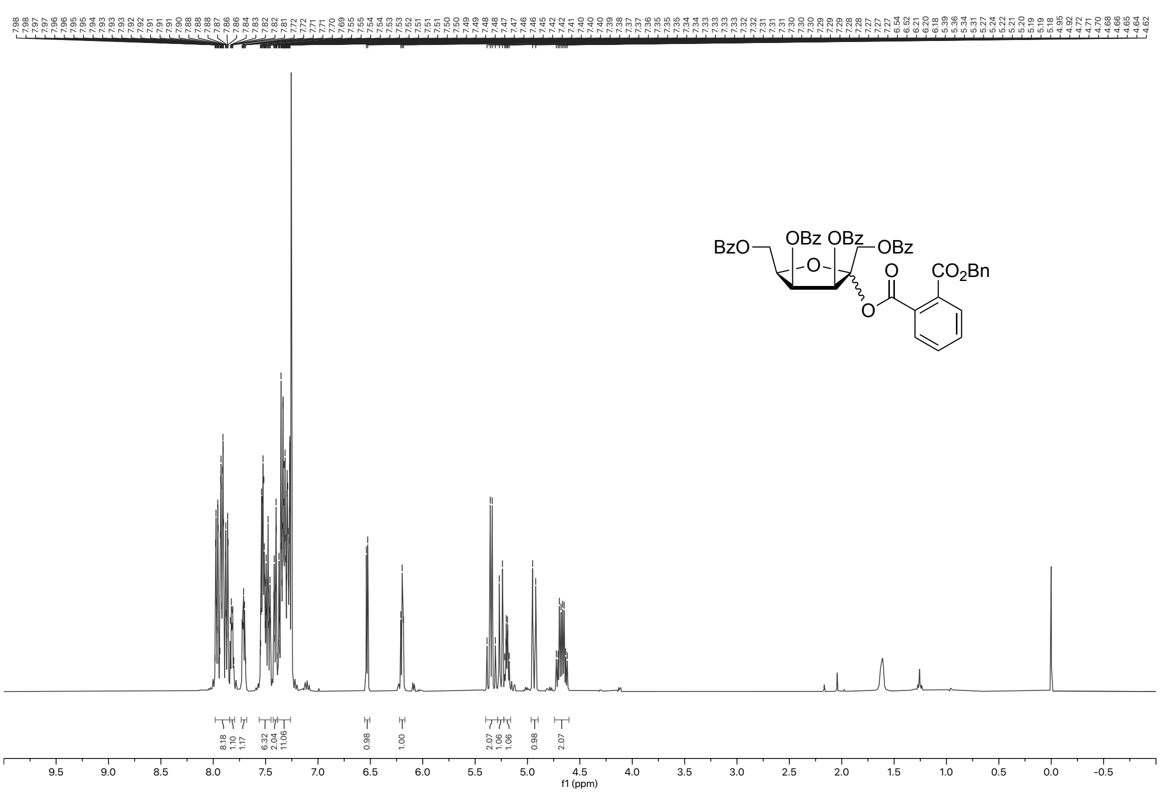

^{13}C NMR of **8** (100 MHz, $CDCl_3$)

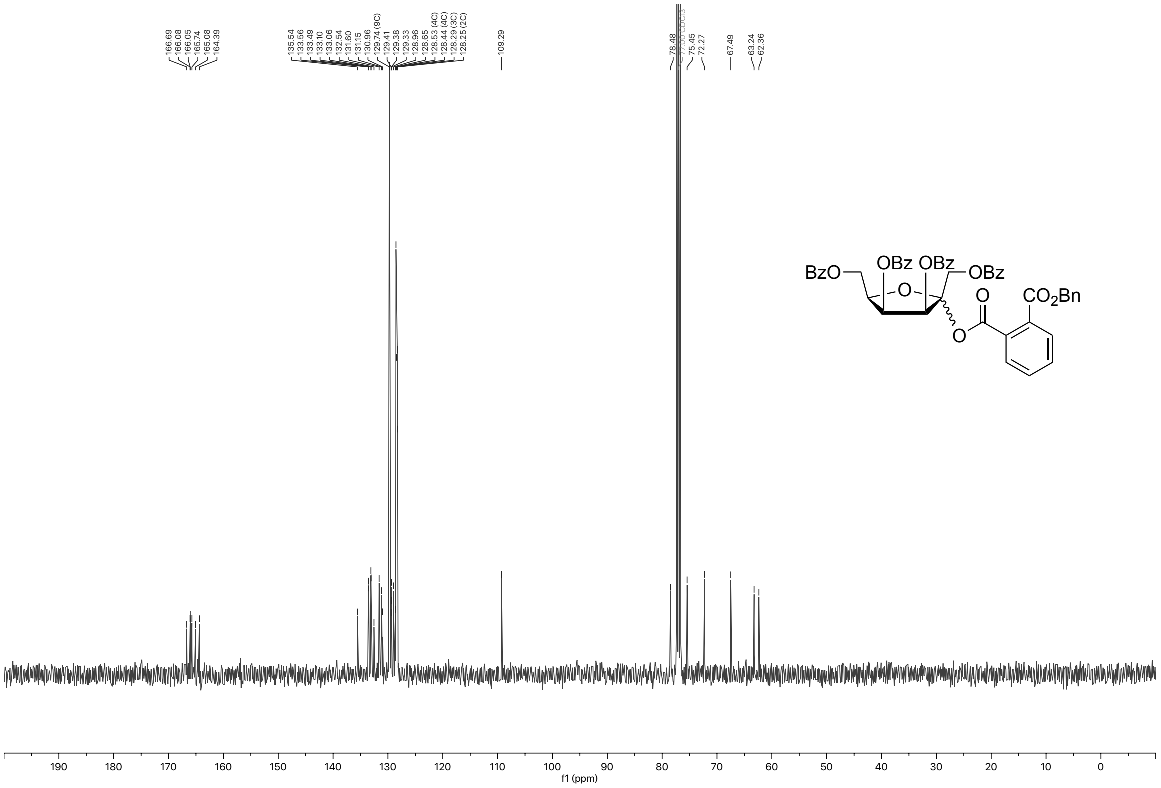

^{1}H NMR of **9a** (500 MHz, $CDCl_3$)

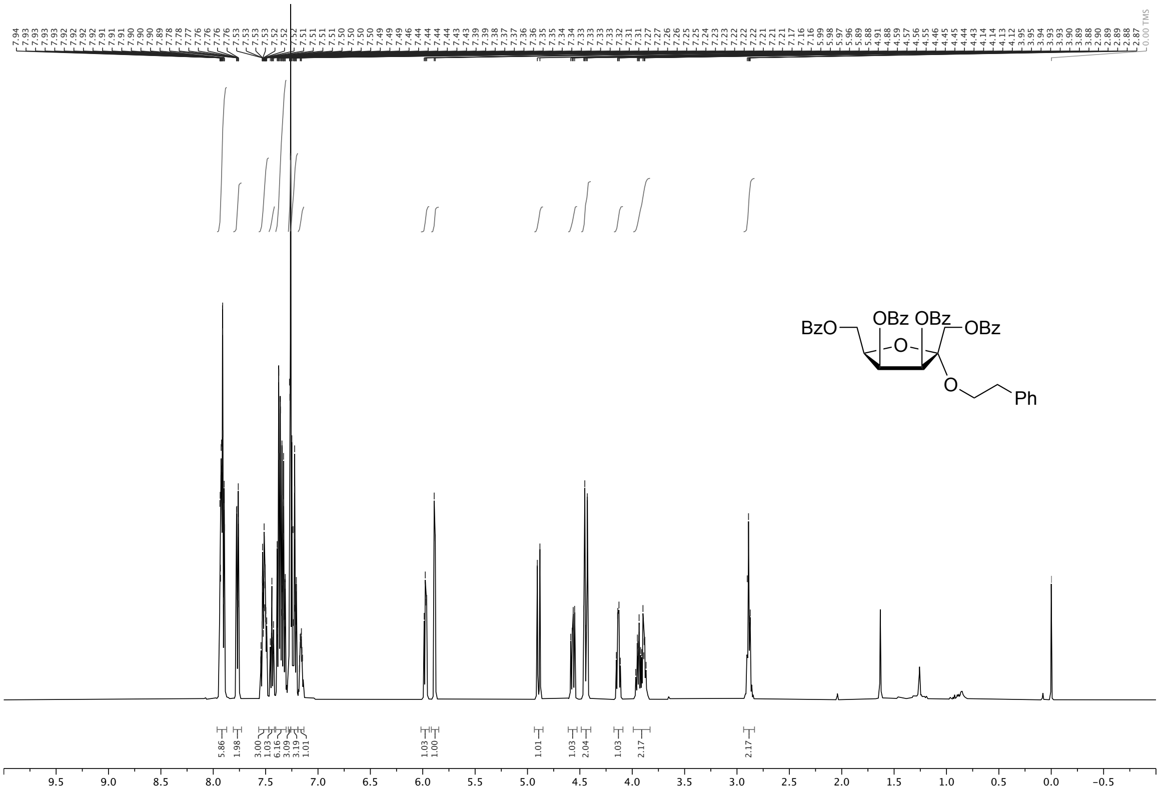

^{13}C NMR of **9a** (126 MHz, $CDCl_3$)

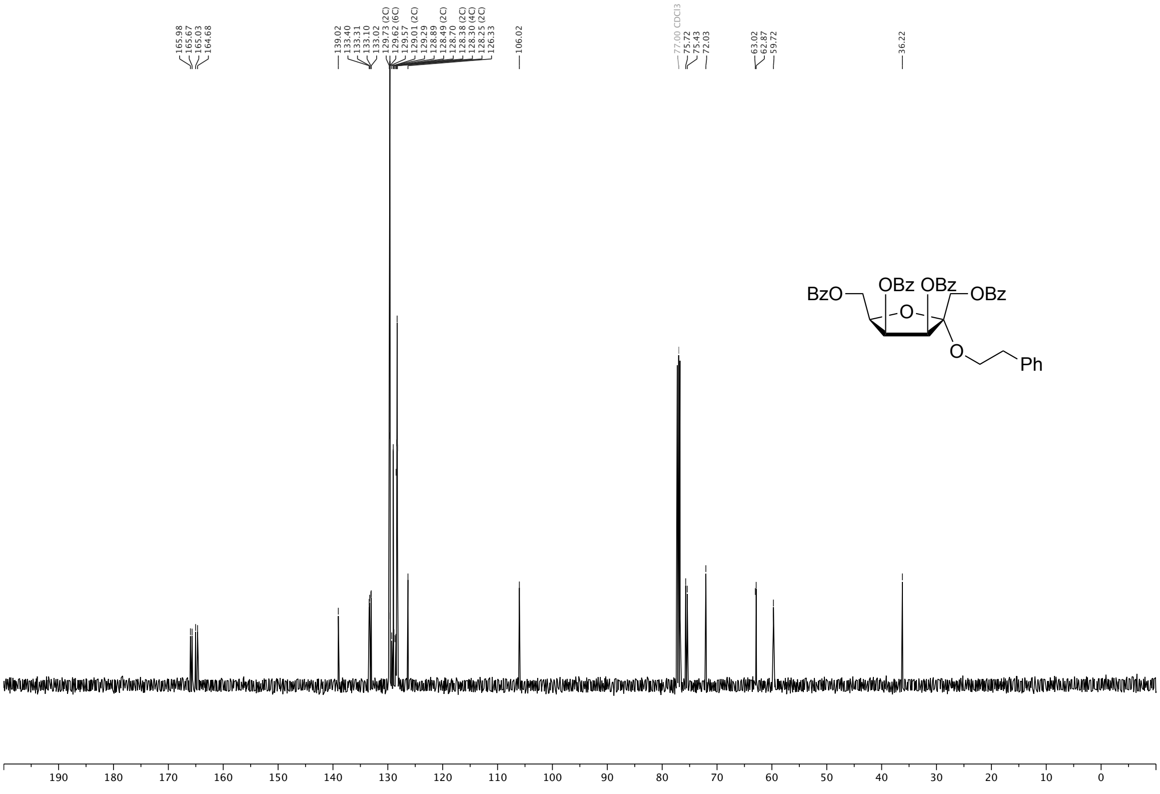

^{1}H NMR of **9b** (500 MHz, $CDCl_3$)

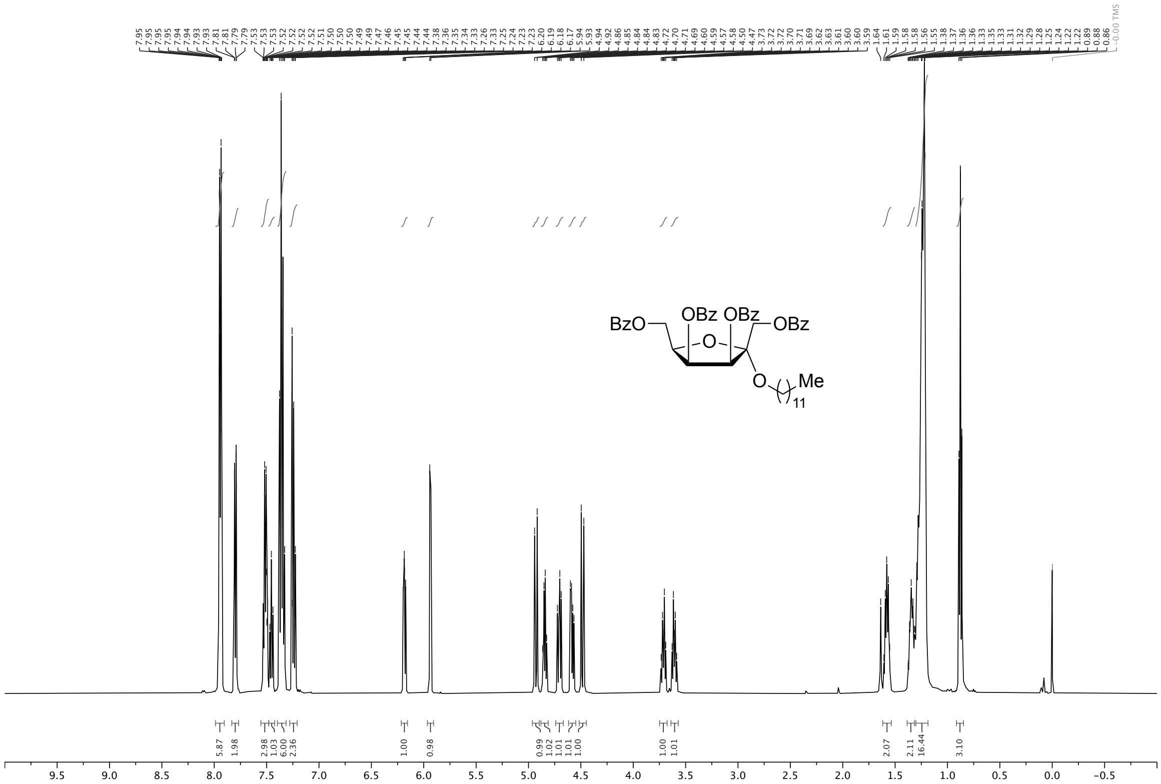

^{13}C NMR of **9b** (126 MHz, $CDCl_3$)

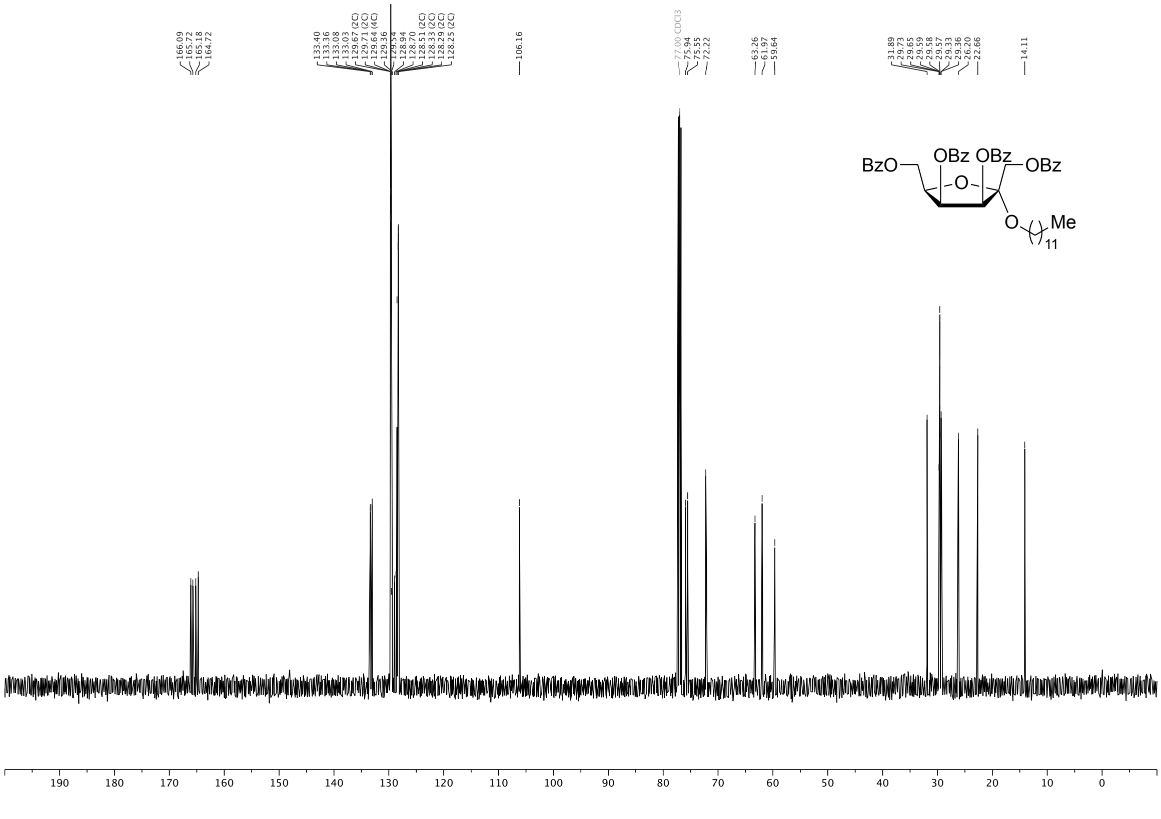

^{1}H NMR of **9c** (400 MHz, $CDCl_3$)

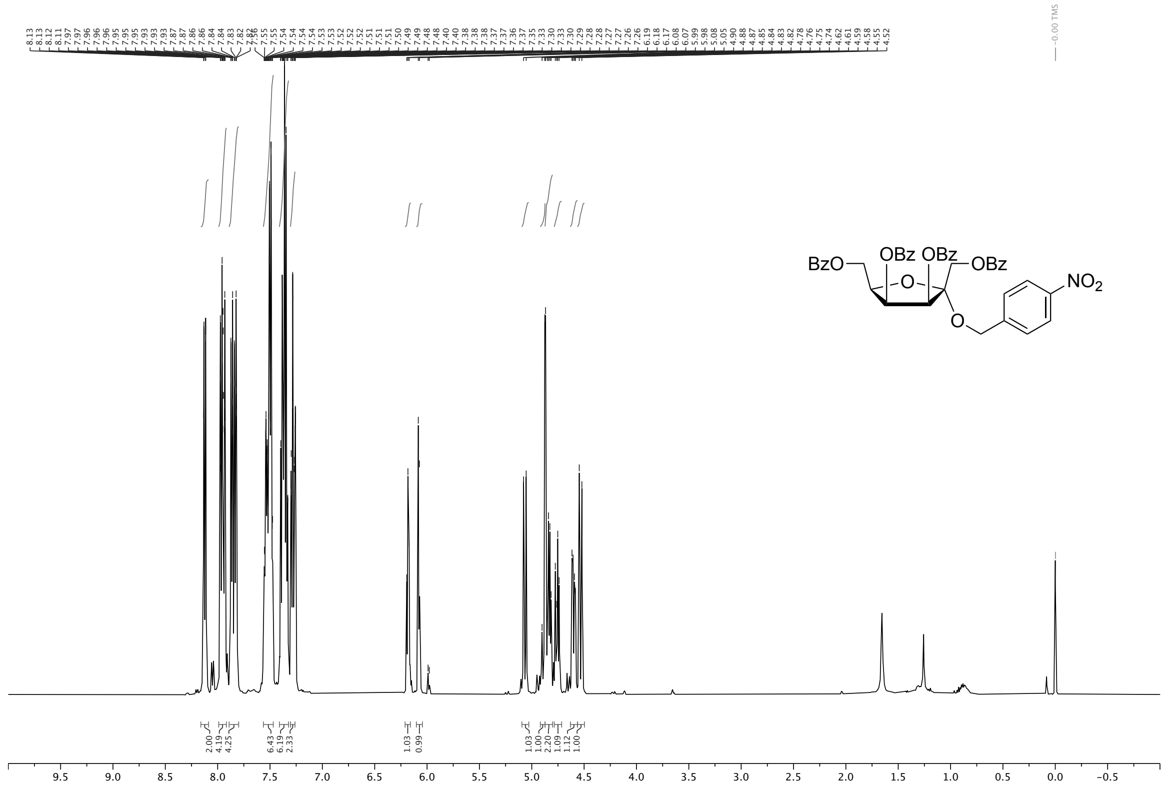

^{13}C NMR of **9c** (100 MHz, $CDCl_3$)

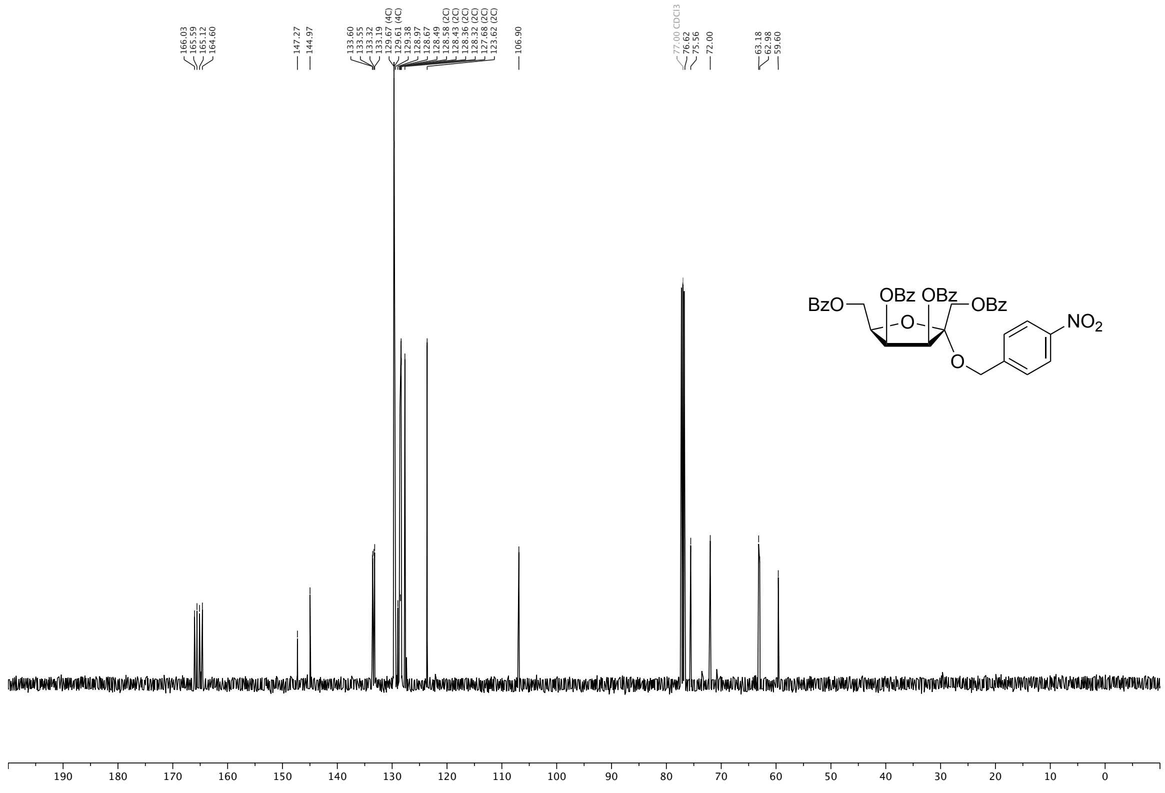

^{1}H NMR of **9d** (500 MHz, $CDCl_3$)

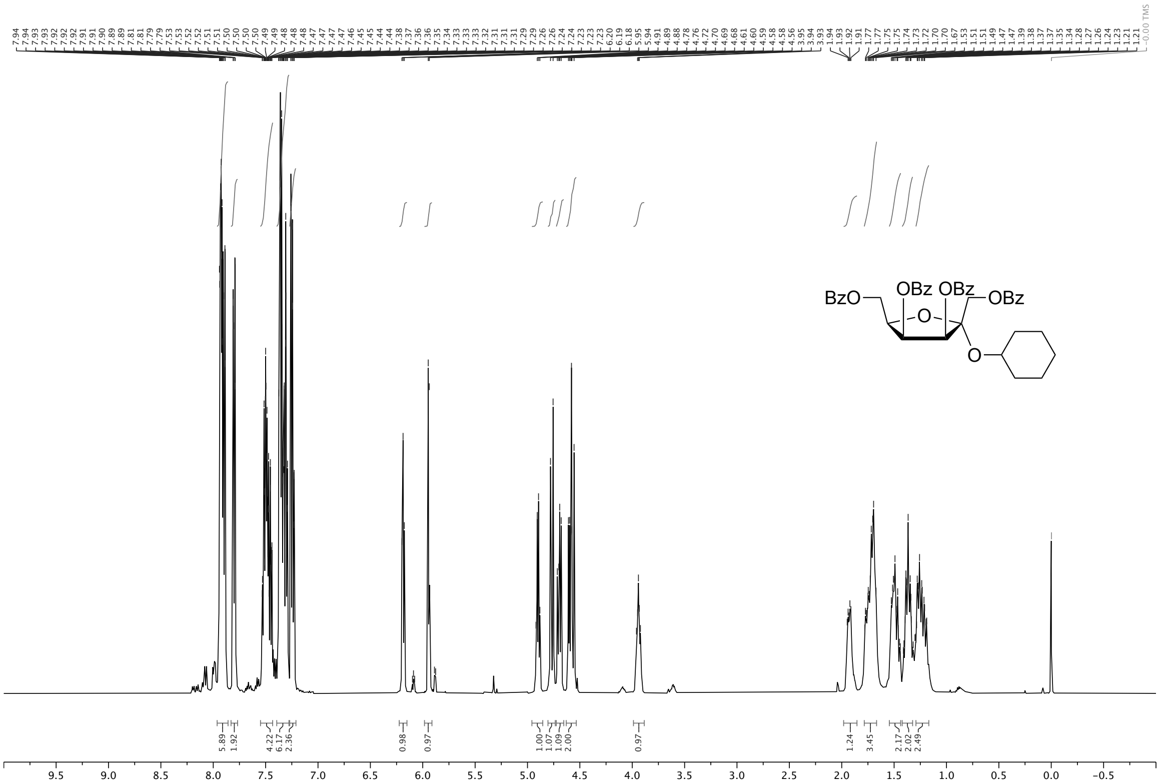

^{13}C NMR of **9d** (126 MHz, $CDCl_3$)

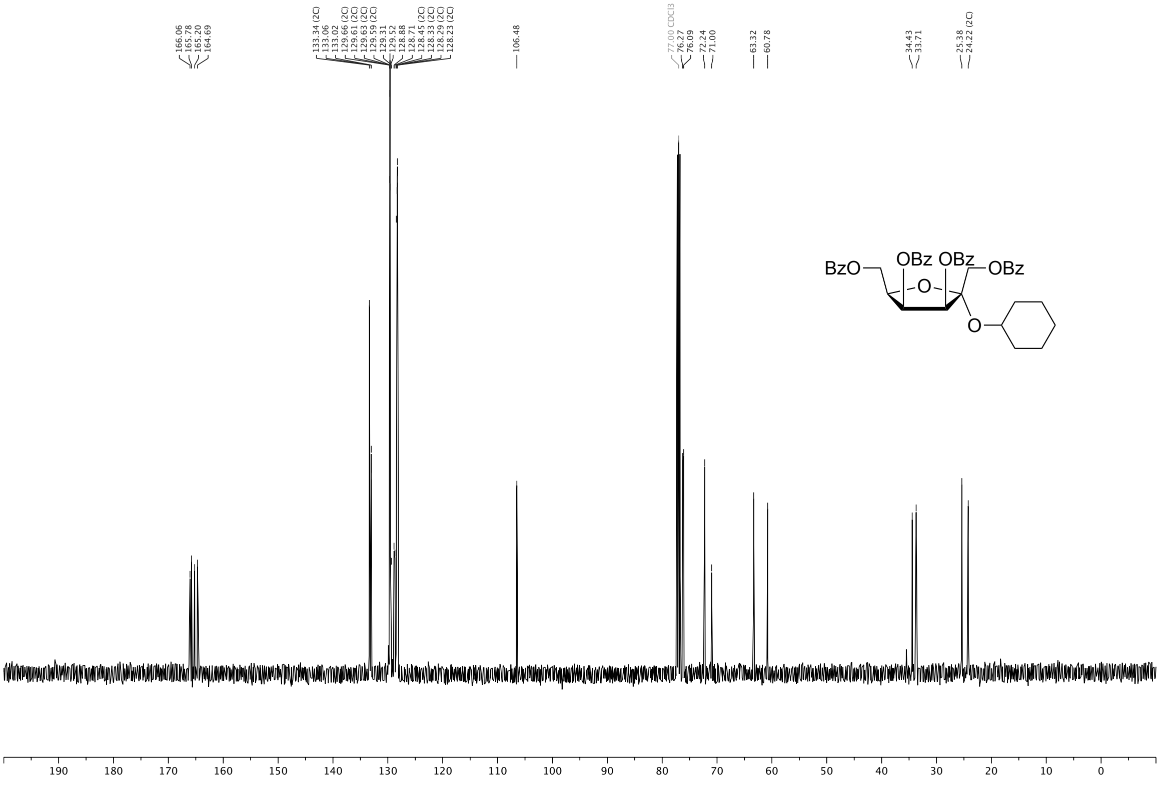

^{1}H NMR of **9e** (500 MHz, $CDCl_3$)

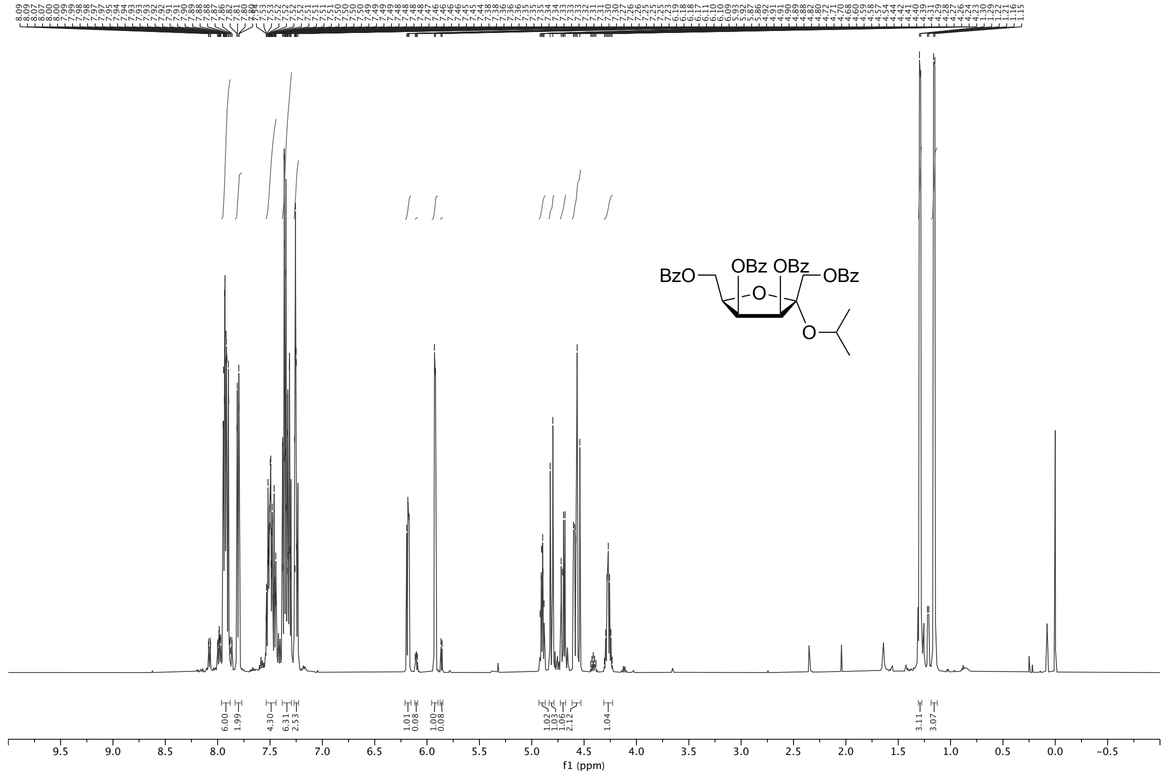

^{13}C NMR of **9e** (126 MHz, $CDCl_3$)

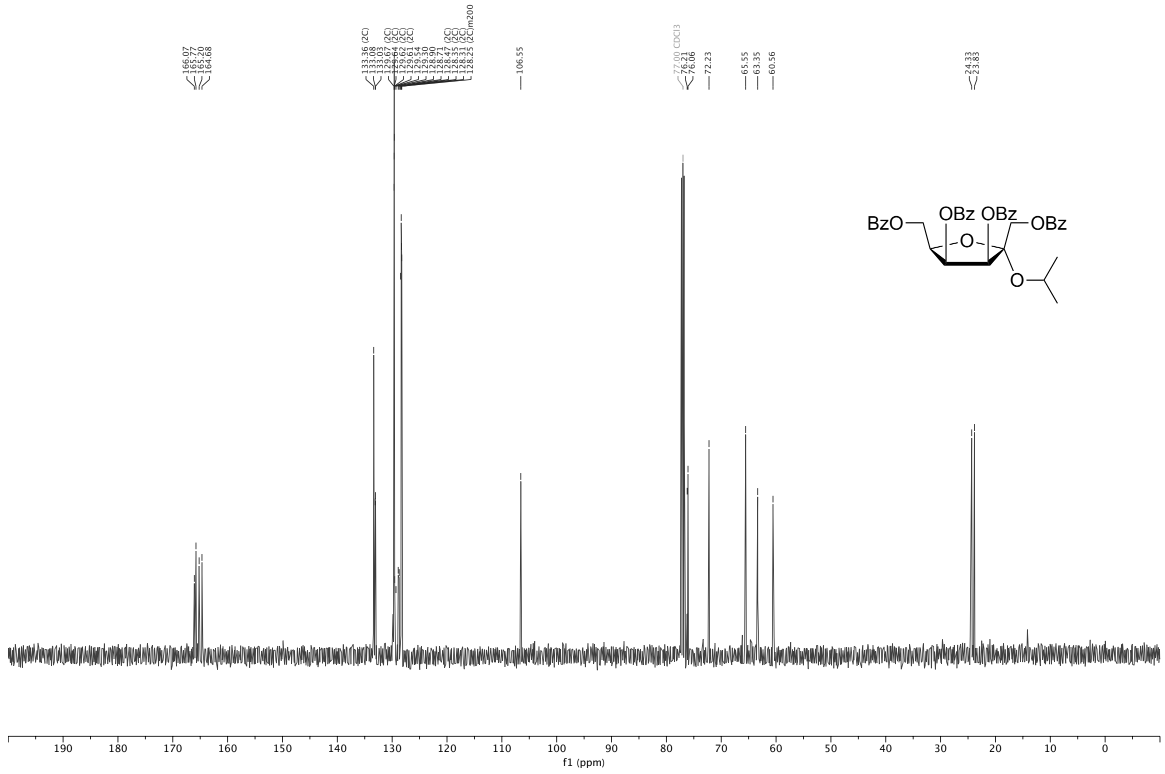

^{1}H NMR of **9f** (500 MHz, $CDCl_3$)

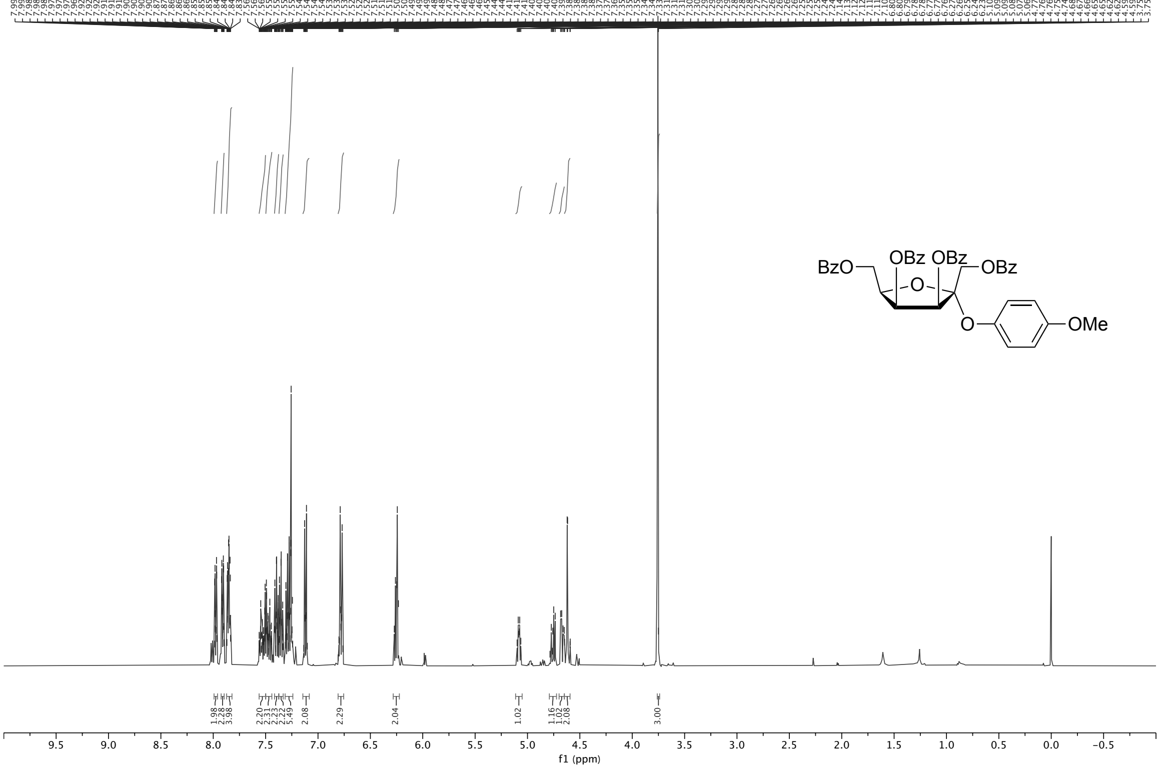

^{13}C NMR of **9f** (126 MHz, $CDCl_3$)

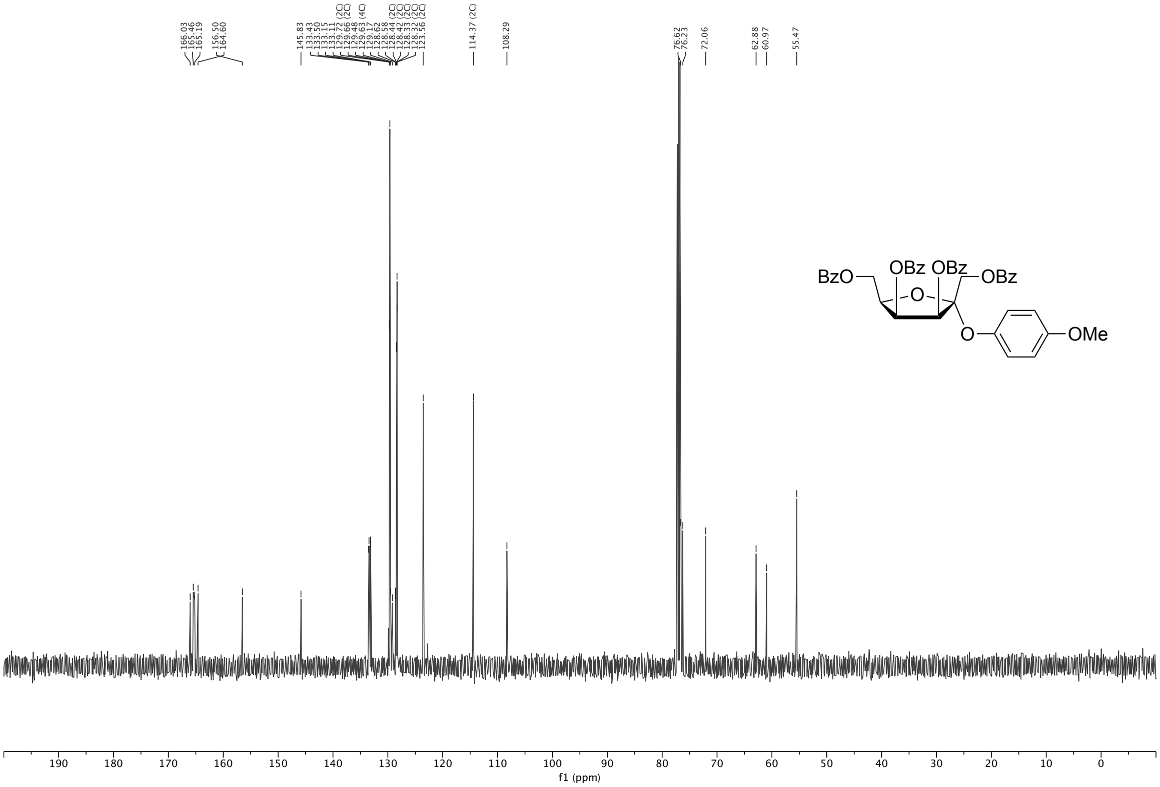

^{1}H NMR of **9gα** (500 MHz, $CDCl_3$)

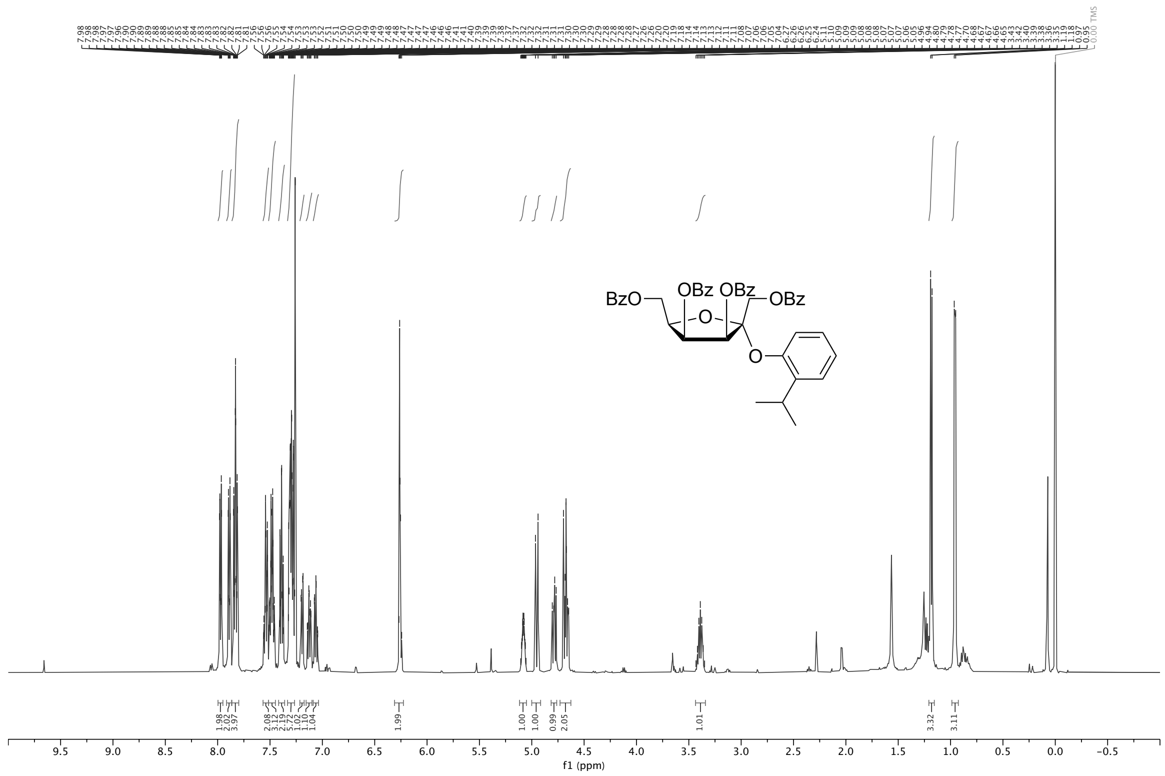

^{1}H NMR of **9gβ** (500 MHz, $CDCl_3$)

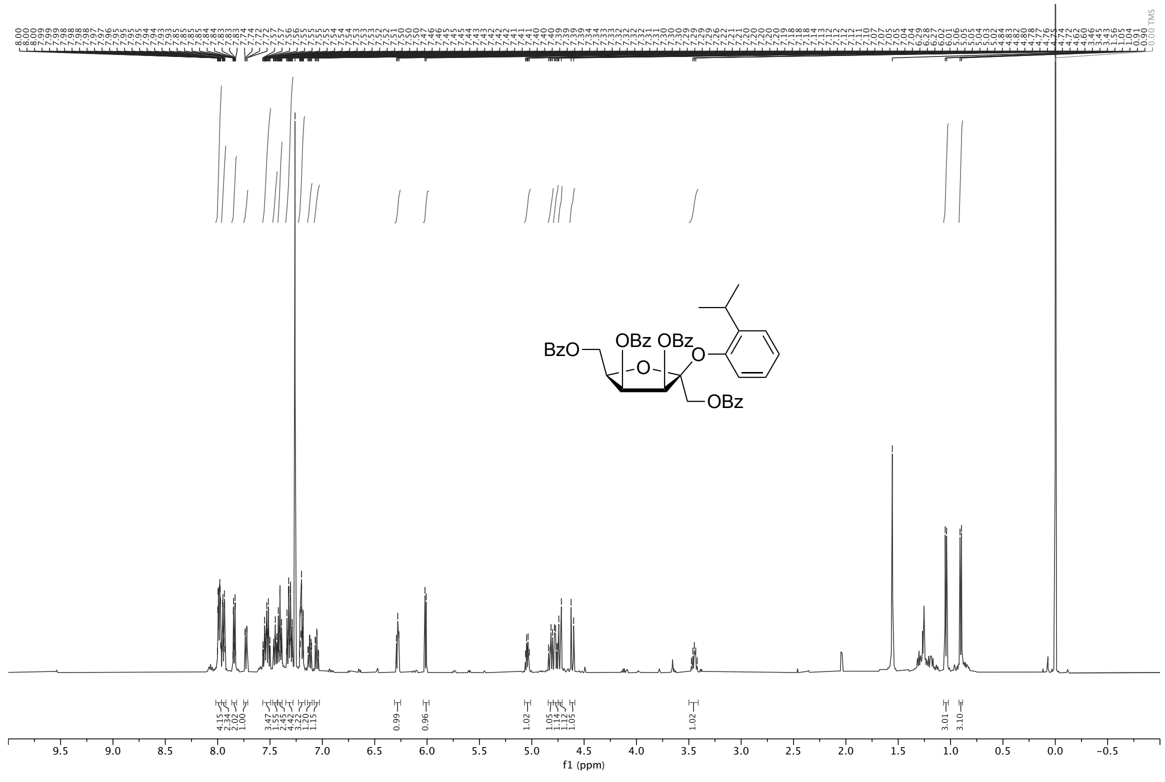

^{1}H NMR of **9h** (500 MHz, $CDCl_3$)

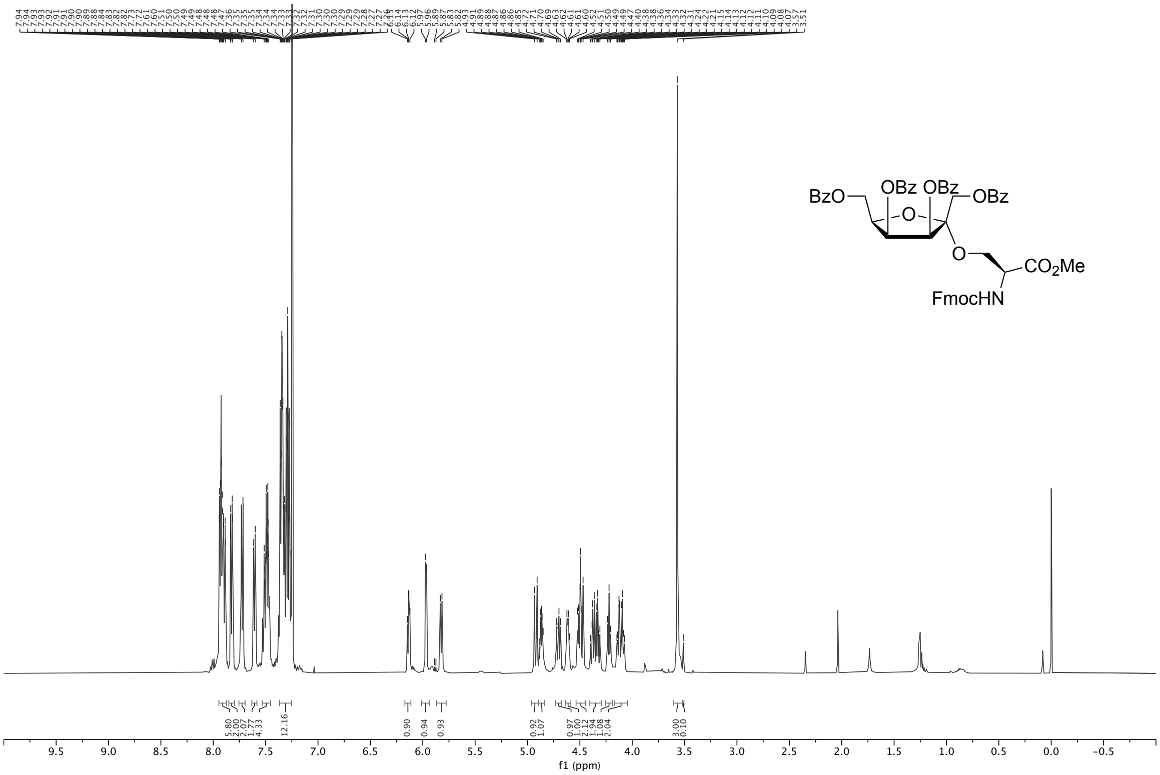

^{13}C NMR of **9h** (126 MHz, $CDCl_3$)

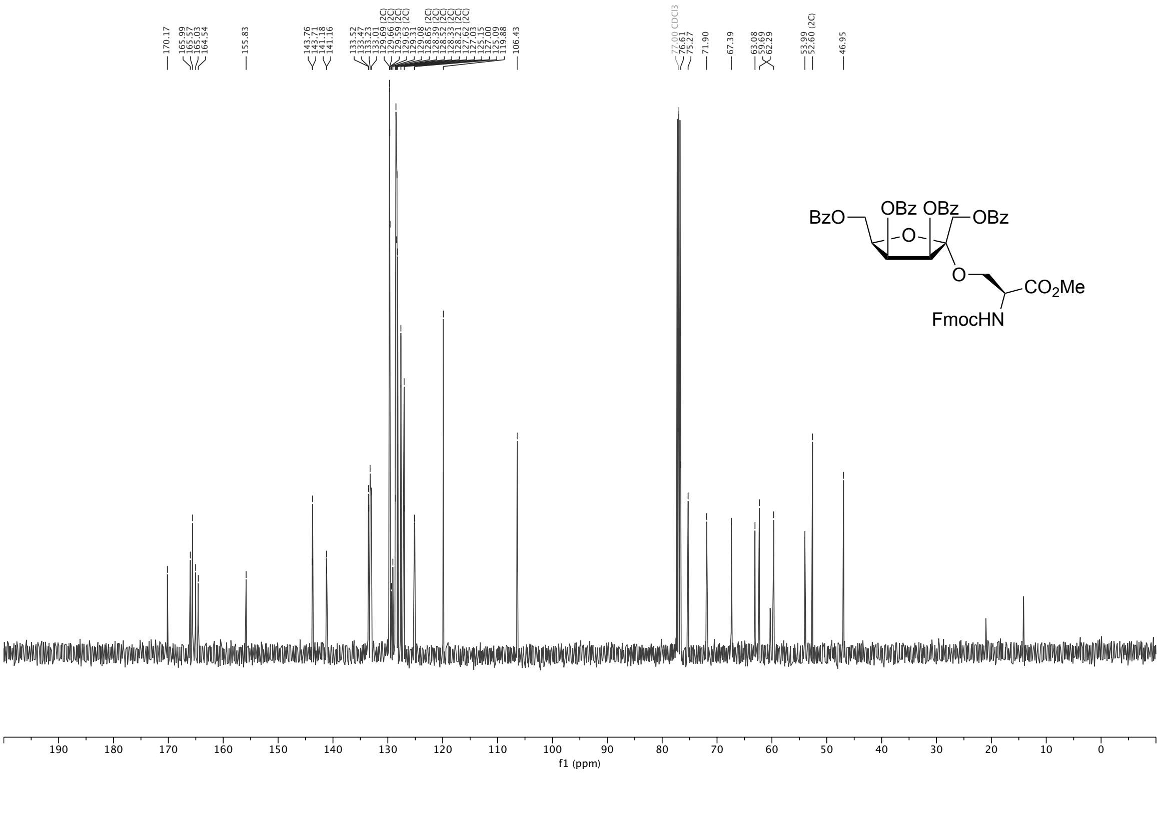

^{1}H NMR of **9i** (400 MHz, $CDCl_3$)

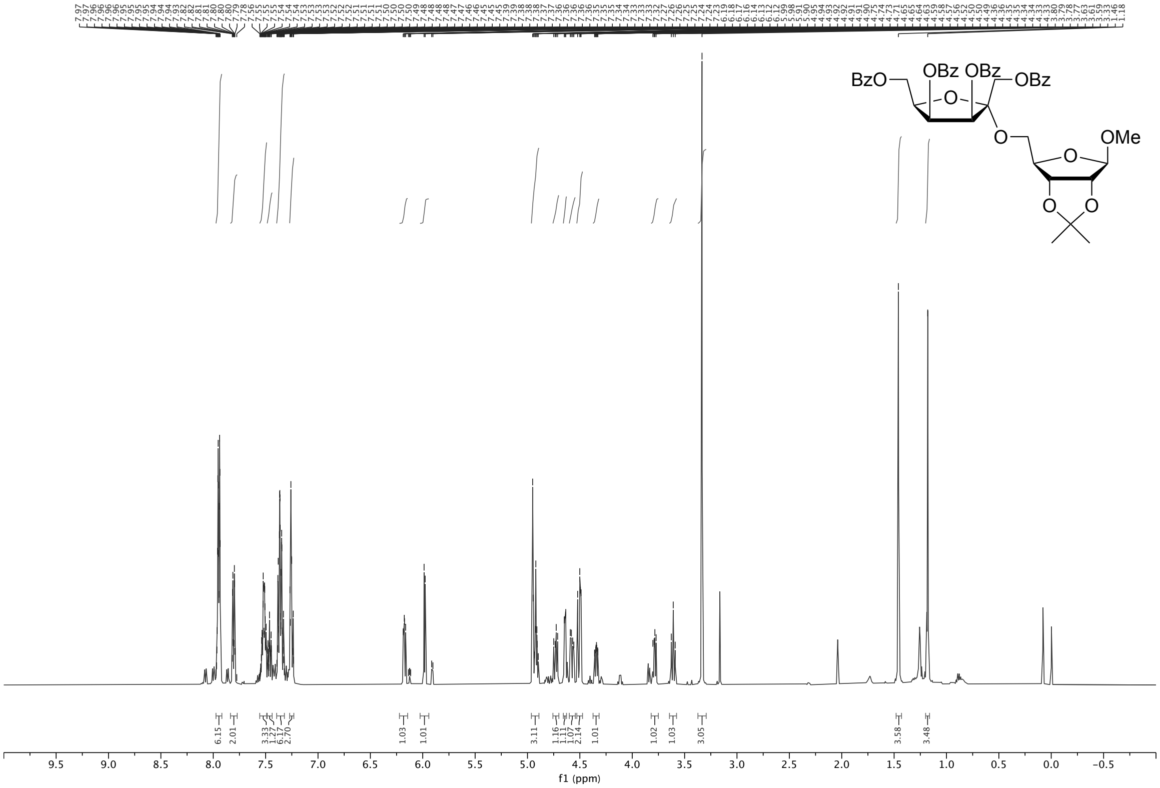

^{13}C NMR of **9i** (100 MHz, $CDCl_3$)

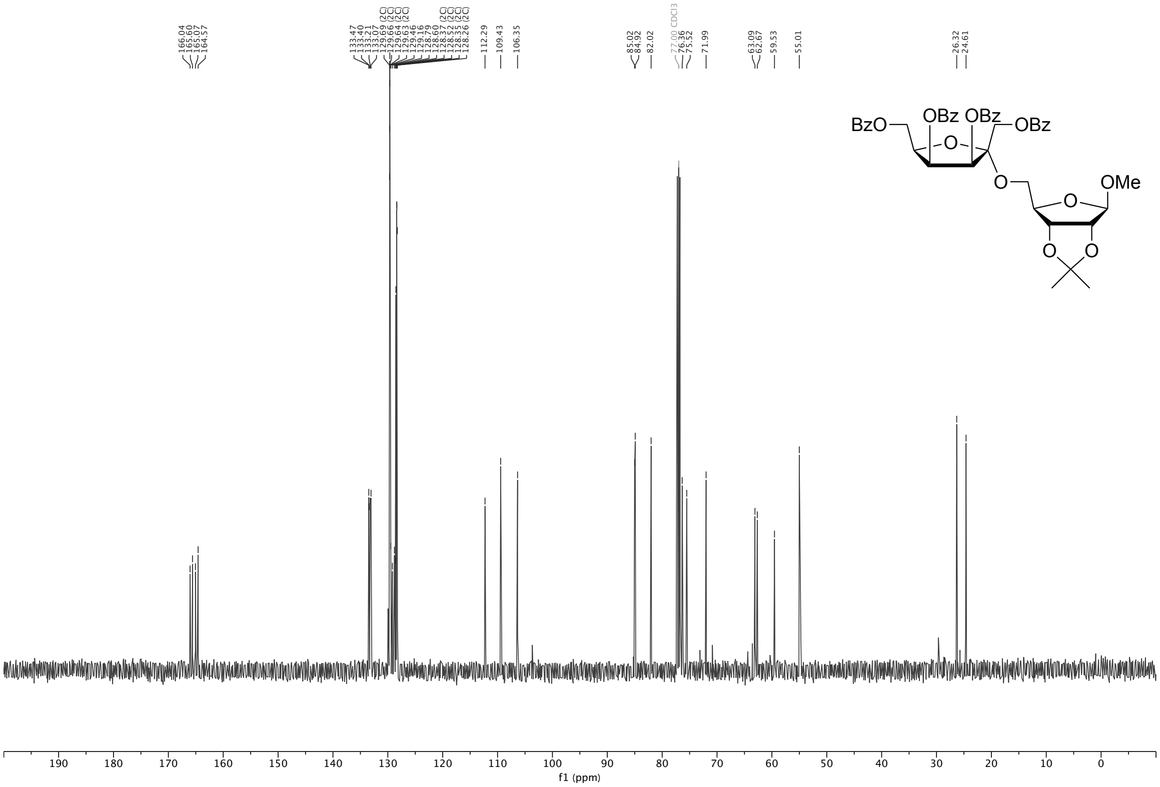

^1H NMR of **9j** (500 MHz, $CDCl_3$)

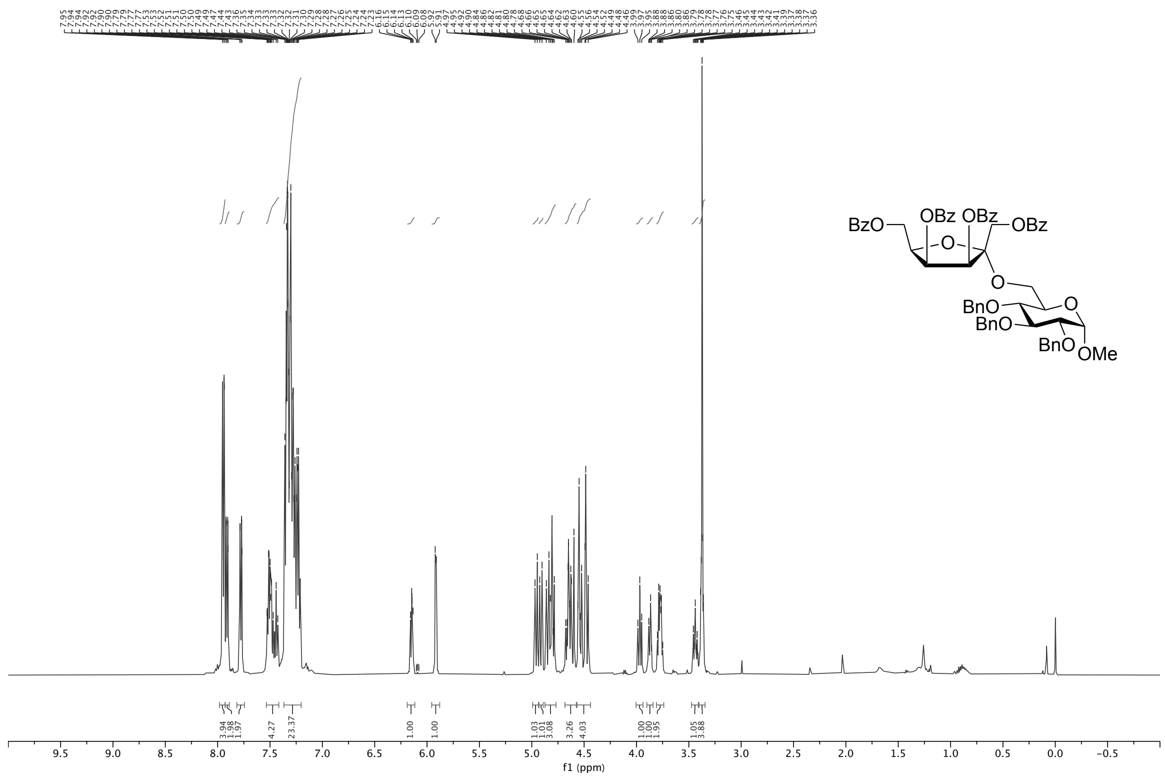

^{13}C NMR of **9j** (126 MHz, $CDCl_3$)

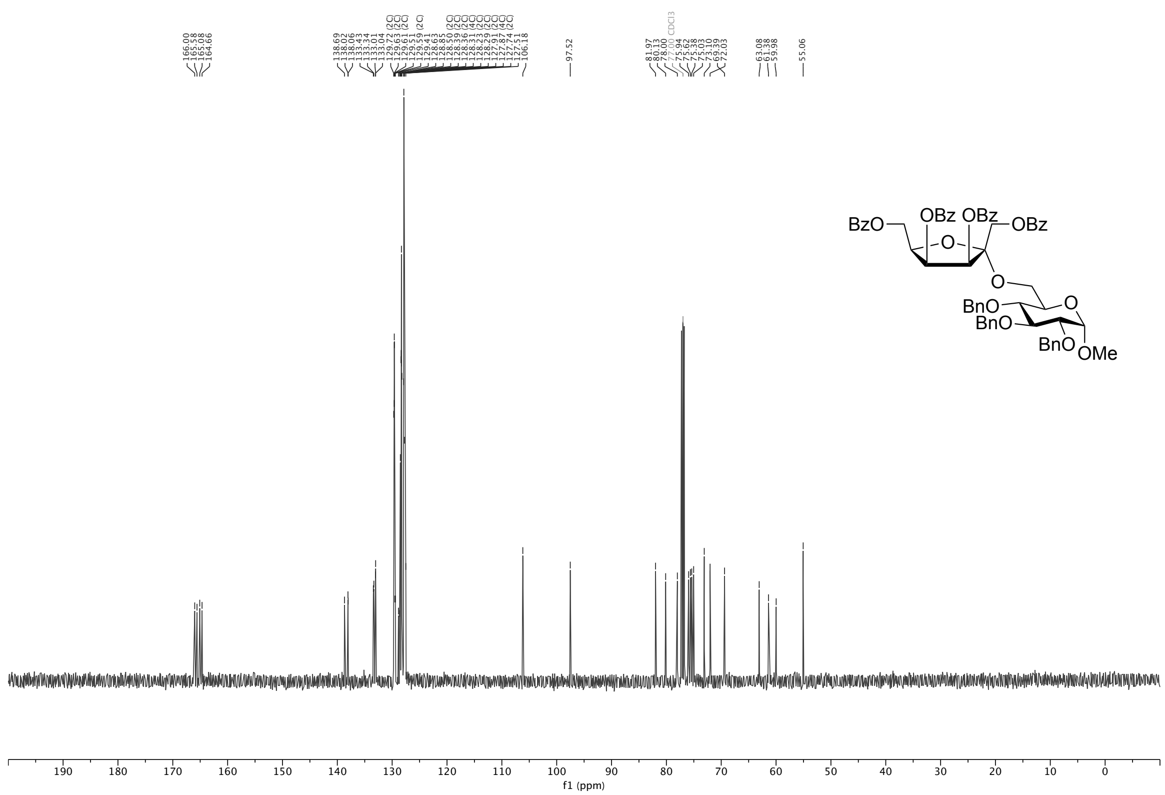

^{1}H NMR of **10** (500 MHz, $CDCl_3$)

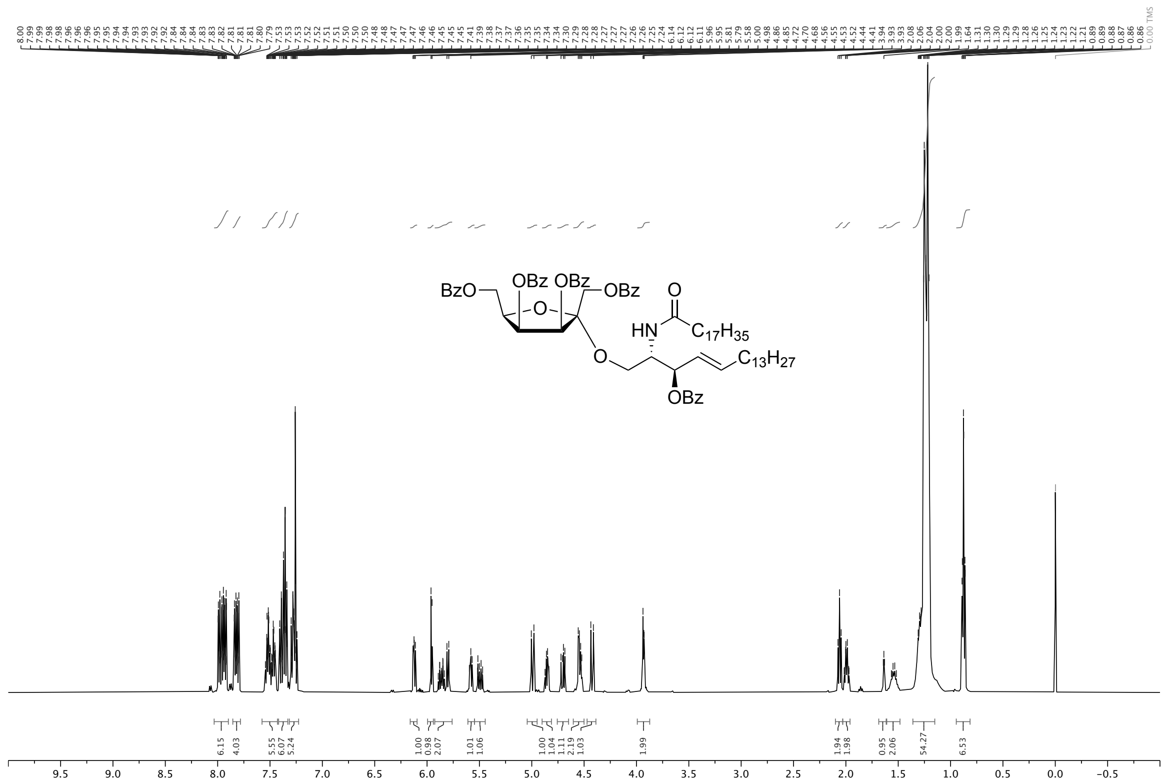

^{13}C NMR of **10** (126 MHz, $CDCl_3$)

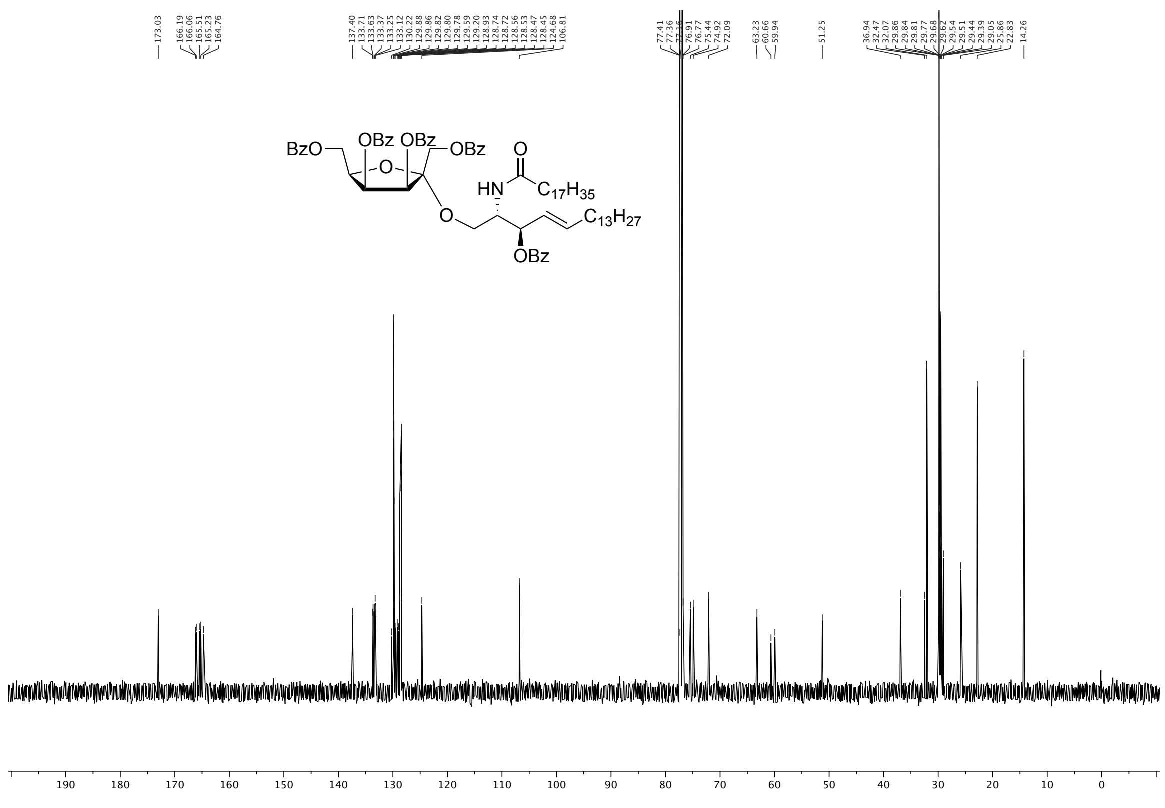

^{1}H NMR of **11** (500 MHz, $CDCl_3/CD_3OD$ = 1:1)

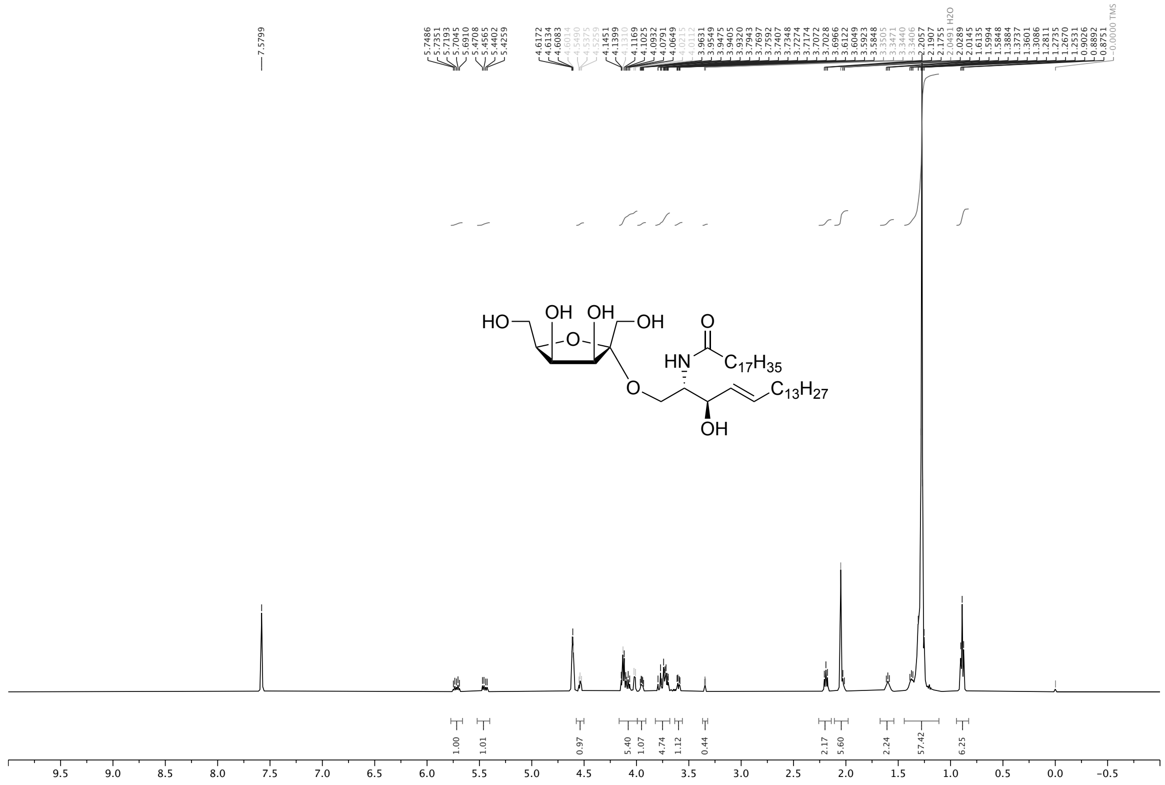

^{13}C NMR of **11** (126 MHz, $CDCl_3/CD_3OD$ = 1:1)

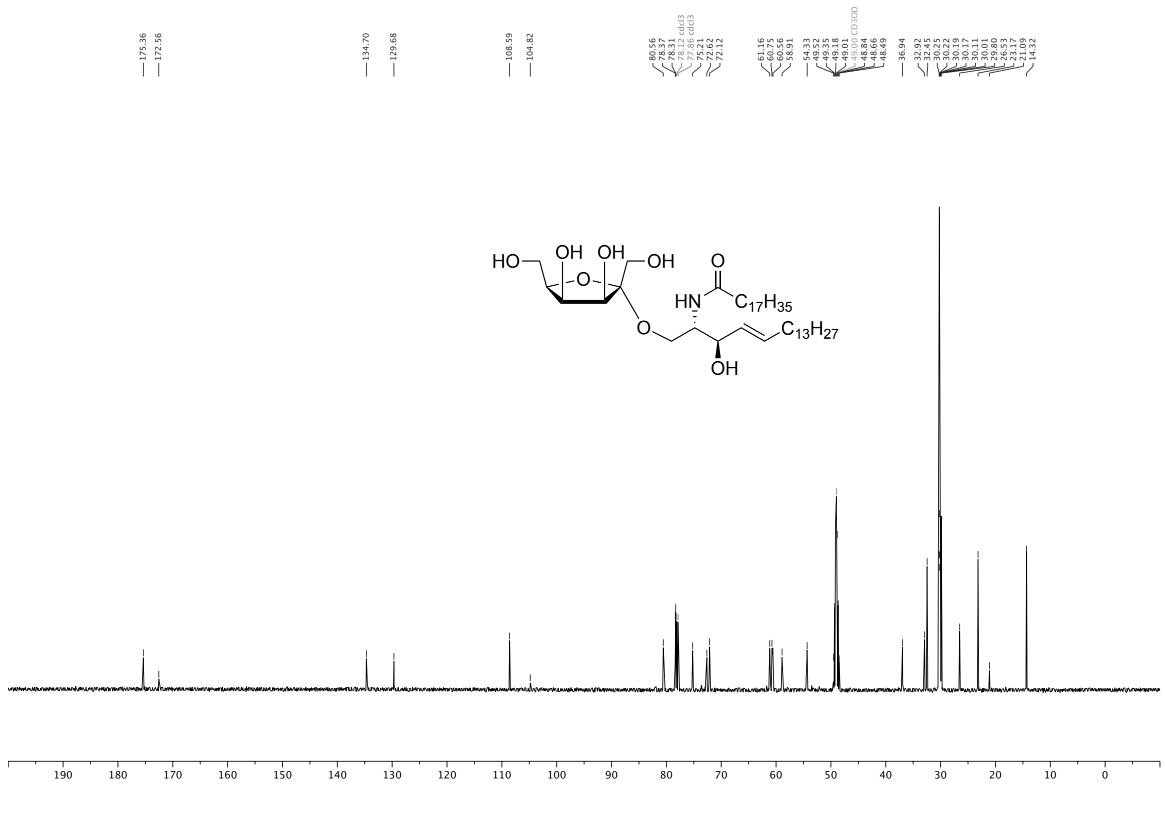

^{1}H NMR of **13**(500 MHz, $CDCl_3$)

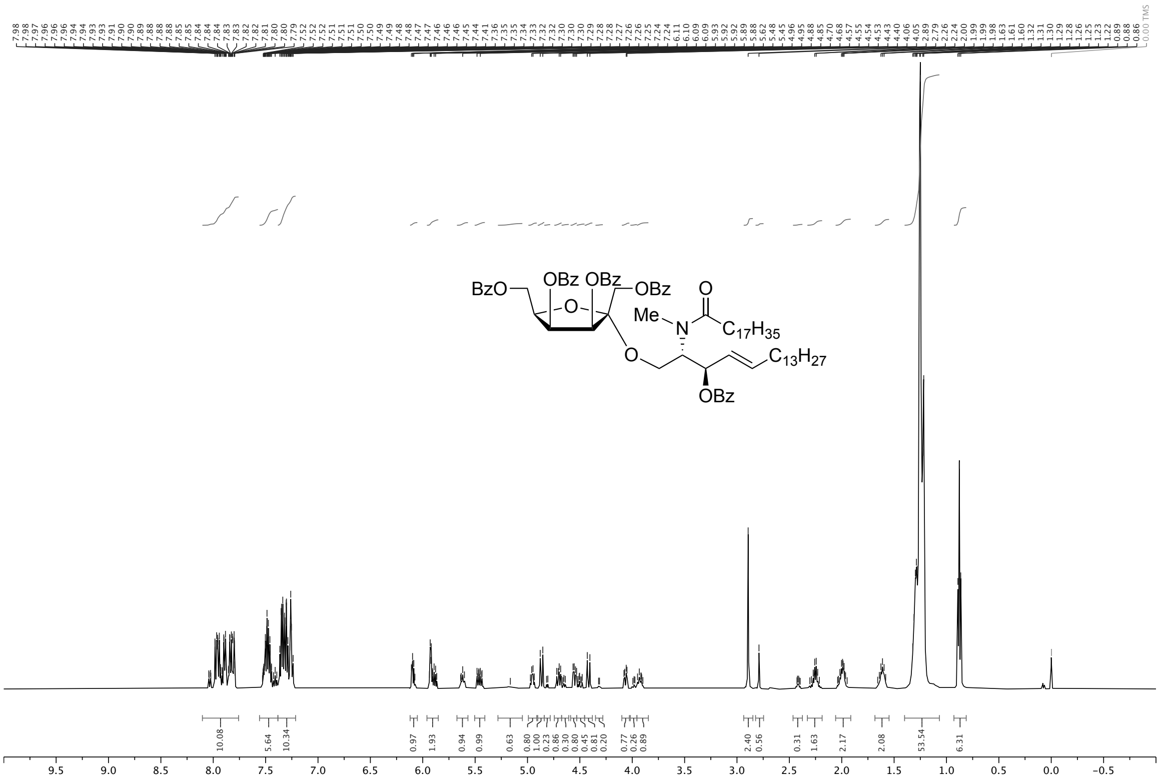

^{13}C NMR of **13** (126 MHz, $CDCl_3$)

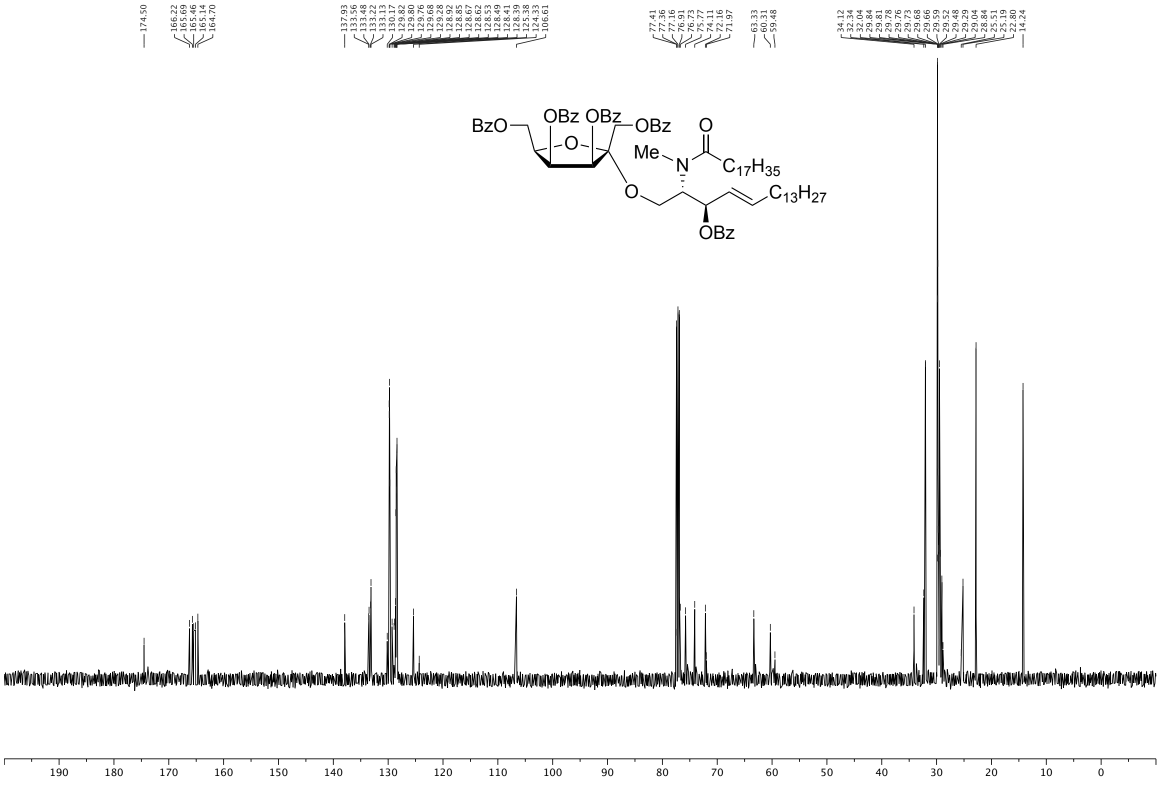

^{1}H NMR of **14** (400 MHz, $CDCl_3/CD_3OD$ = 1:1)

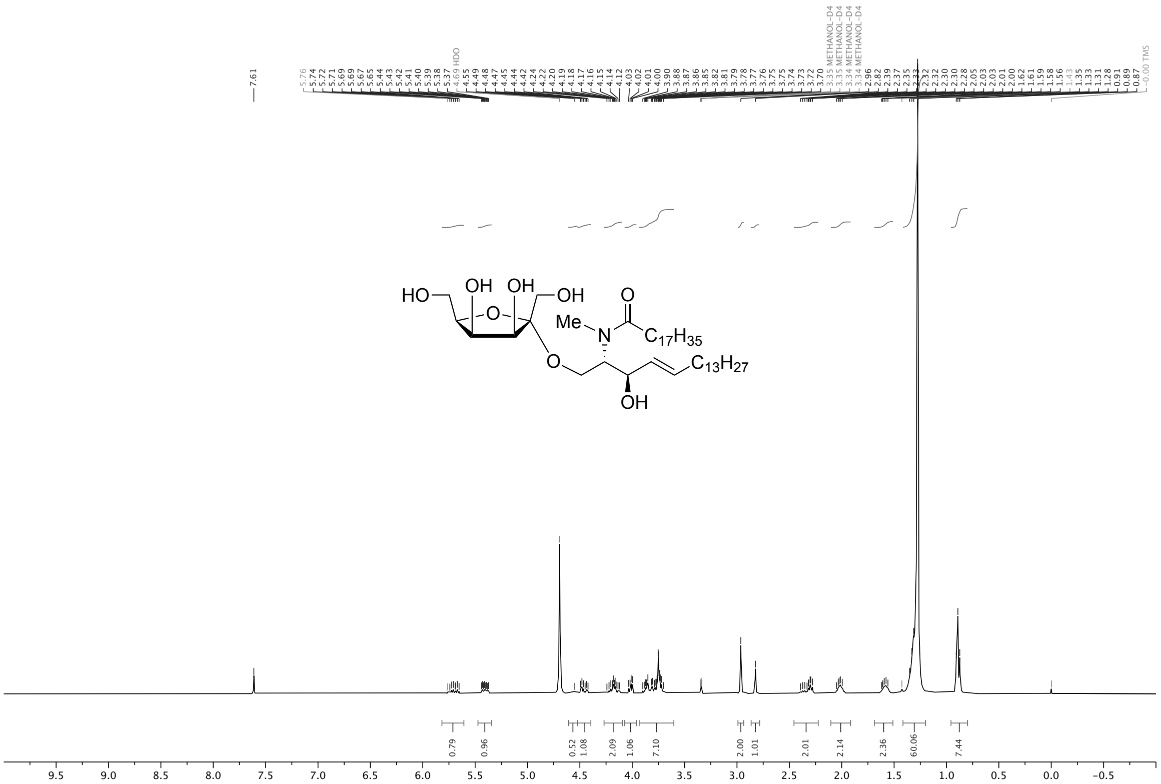

^{13}C NMR of **14** (100 MHz, $CDCl_3/CD_3OD$ = 1:1)

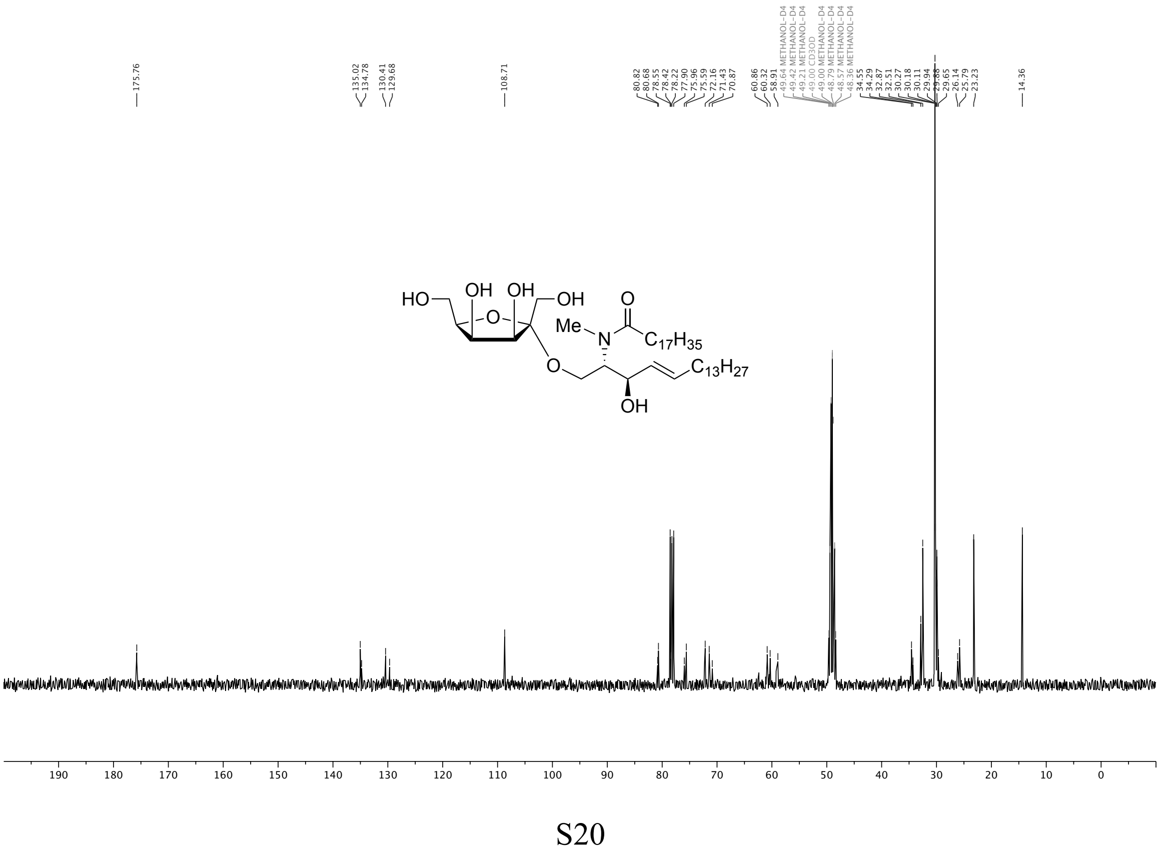

Supplement: Supplementary file 1 [file ijms-26-08459-s001.zip › ijms-3822281-supplementary.pdf]
